# Reversing cardiac hypertrophy and heart failure using a cardiac targeting peptide linked to miRNA106a

**DOI:** 10.1002/ctm2.70432

**Published:** 2025-08-17

**Authors:** Ming Lu, Siqi Cai, Kyle Korolowicz, Claire Deng, Kaan Taskintuna, Gerard Ahern, Raymond B. Yurko, Kazi R. Islam, Bryn Schoonover, Jack B. Lopuszynski, Hongkun Wang, Maliha Zahid, G. Ian Gallicano

**Affiliations:** ^1^ Department of Biochemistry and Molecular Biology Georgetown University Medical Center Washington District of Columbia USA; ^2^ Department of Oncology Lombardi Comprehensive Cancer Center Washington District of Columbia USA; ^3^ Department of Pharmacology Georgetown University Medical Center Washington District of Columbia USA; ^4^ Health Science Core Research Facilities Peptide and Peptoid Synthesis Core Facility University of Pittsburgh Pittsburgh Pennsylvania USA; ^5^ Dept. of Cardiovascular Medicine Mayo Clinic Rochester Minnesota USA; ^6^ Department of Biostatistics Bioinformatics, and Biomathematics Georgetown University Washington District of Columbia USA

**Keywords:** cardiac targeting peptide, heart failure, miRNA therapy, NF‐kB

## Abstract

**Background:**

One in five adults aged 40 will develop heart failure (HF) during their lifetime. Risk factors (e.g., hypertension, diabetes mellitus, coronary artery disease, etc.) lead to structural and functional changes in cardiomyocytes, resulting in HF. At the cellular level, these changes consist of mis/over‐expression of genes that regulate cardiac identity (e.g., CamK2δ, PKC, Stat3, etc.). The current paradigm for treating HF is pharmacological or device‐based intervention; however, with few exceptions, the condition worsens with time. We are proposing to implement a change in HF treatment, shifting from a drug‐centric system to a cardiac target‐specific molecular approach that would reverse hypertrophy and adverse remodelling of affected cardiomyocytes.

**Methods:**

A cardiomyocyte targeting peptide (CTP) was reversibly linked to miRNA106a for delivery to a mouse model of HF. Reversal of morphological, signalling, and physiological HF parameters was measured. Additionally, CTP‐miRNA106a was introduced into a human cardiomyocyte cell line to identify mechanism(s) at play for reversing HF characteristics (e.g., hypertrophy).

**Results:**

Bio‐distribution studies showed that intravenously injected CTP‐miRNA106 delivered its cargo specifically to the heart within 30 min, followed by clearance of CTP from the heart to the kidneys, and to a lesser extent, the liver by 35 h with persistence of miRNA106a in cardiomyocytes until day 7 (the latest tested time‐point). CTP‐miRNA106a reversed angiotensin2/isoproterenol‐induced hypertrophy in 90% of the treated mice. We also identified two potential HF intracellular signalling pathways/mechanisms (PLCβ1/PKC/IP3 and NF‐κB) targeted by CTP‐miRNA106a that could benefit many pathophysiologies underlying HF, including inflammation.

**Conclusions:**

CTP‐miRNA106a, a first‐of‐a‐kind cardiac‐specific drug, downregulates genes involved in cardiac hypertrophy and inflammation through the PLCβ1 and CamKIIδ kinase pathways. CTP delivery of miRNA106a cargo is specific to cardiomyocytes both in vitro and in vivo, and once delivered, many HF parameters, including hypertrophy, are reversed.

**Key points:**

- Cardiac Targeting Peptide (CTP) delivers, specifically to the heart, reversibly linked miRNA106a.
- MiRNA106a targets genes in various pathways known to cause heart failure when they are over/hyper activated returning their expression to normal levels.
- In vivo analyses using a mouse heart failure model resulted in reversal of heart failure parameters in 19/20 mice.

## Graphical abstract

Molecular pathways involved with heart failure are complex. We generated a cardiac targeting peptide (CTP) linked to miRNA106a, a first‐of‐a‐kind cardiac‐specific drug, to downregulate genes involved in hypertrophy and inflammation, specifically PLCβ1 and CamKIIδ pathways. CTP delivers miRNA106a cargo specifically to cardiomyocytes in vitro and in vivo.

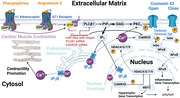

## 1 INTRODUCTION

Cardiovascular disease (CVD) remains the leading cause of morbidity and mortality in Western countries.^1^ Heart failure (HF) patients represent about 5% of CVD patients, affecting approximately 23 million people globally, including 6 million in the USA.^2^ Current drug therapy approaches do not resolve the underlying molecular processes that result in stressed myocardium, which are common to many types of HF. Given its growing prevalence, successful treatments that address dysfunctional myocardium at the cellular/molecular level need to be developed for clinical application. In addition, for any newly developed treatment to be fully effective in heart tissue, it needs to be targeted directly to the heart so that all other organs are spared adverse events. Here, we present the effects of a cardiac targeting peptide (CTP) that delivers, specifically to the heart, a reversibly linked microRNA that targets genes known to cause HF when misexpressed.

A thorough review^3^ recently discussed the intracellular signalling similarities and differences between many types of HF. Key molecular players that can be found include G‐protein‐coupled receptors (GPCRs) and their hormone ligands (angiotensin II, endothelin 1, α‐adrenergic receptors and β‐adrenergic receptors); signalling kinases (p38, ERK1/2, JNK, CAMKII, PKC, PKG, PKA, mTORC1 and AMPK), epigenetic modulators (NFAT, MEF2, GATA4, class II HDACs and Hippo) and mechanosensitive plasma‐membrane cation channels (TRPC and TRPV). The question remains as to why drug regimens that target various members of these specific pathways are beneficial for some types of HF but not others. A potential answer to this question may be enshrouded within a few recent publications,^4^, ^5^ which have suggested the establishment of centralized “hubs” of intracellular signals that become over‐ or misexpressed by overstimulated hormonal receptors (e.g., AT1R, β1AR, etc.). Consequently, it is possible that current drugs that affect one signalling hub may not affect others, resulting in the treatment dichotomy surrounding different forms of HF.

Illustrated in previous studies,^4^, ^5^ this hub concept identifies the delta isoform of Ca^2+^/calmodulin‑dependent protein kinase II (CaMKIIδ‐present in cardiomyocytes) as one primary signalling molecule that regulates multiple cellular pathways. It functions by phosphorylating proteins involved in excitation–contraction coupling and relaxation events in the heart, cardiomyocyte apoptosis, and transcriptional activation of genes related to cardiac hypertrophy, inflammation, and arrhythmias.^6^ If left uncorrected, hyperactive/overexpression of CaMKIIδ‑regulated changes culminate in a dysfunctional myocardium and HF. Alternatively, inactivation of CaMKIIδ is known to be protective against a variety of stress‐induced cardiac dysfunctions.^6^, ^7^, ^8^ Though much has been learned about HF regarding the pathological remodelling of the heart and the role of CaMKIIδ, there remains no specific, targeted inhibitor of CaMKIIδ expression for therapeutic purposes.^9^

Delving further into the literature, other HF hub signalling mechanisms have begun to emerge including the Phospholipase Cβ1/protein kinase C/inositol triphosphate (PLCβ1/PKC/IP3) pathway, protein kinases A and G (PKA, PKG), and nuclear factor κB (NF‐κB) pathway ^10^, ^11^ While these signalling proteins have been targeted for reversing HF, most studies have targeted them individually resulting in limited effects. One example of this was found in a recent editorial and an original paper that questioned the efficacy of various PKC antagonists for treating HF in humans. Significant differences are observed, especially when comparing the animal models that inspired the human clinical trials.^12^, ^13^ While they qualified the current discordant human data as possible species differences, dosage, or timing and duration of PKC inhibition/reduction, another potential flaw in PKC antagonist research may have been due to overlooking the hierarchical components of the pathway: activation of PLCβ1 by AT1R. PLCβ1 cleaves phosphoinositol bisphosphate into diacylglycerol (DAG) and inositol triphosphate (IP3), with the DAG activating PKC and IP3 receptors internally to release calcium into the cytosol. PKC antagonists do not inhibit the IP3 side of the bifurcated pathway. Thus, a take‐home message from the combined findings in the literature and our laboratory is that for an HF drug to be truly effective, it must target more than one HF‐causing hub component.

MiRNAs are small, noncoding RNA molecules (containing ∼22 nucleotides that function to silence mRNA through a 7–8 base seed sequence, leading to post‐transcriptional regulation of gene expression.^14^, ^15^, ^16^, ^17^, ^18^ Previously, we demonstrated the importance of miRNAs 17, 20a, 93, and 106a (all have the same seed sequence) in regulating genes involved in cardiac cell development.^16^, ^19^, ^20^ Those findings led to the idea that one or more of these miRNAs could be used to reestablish cardiac identity in cardiac cells undergoing hypertrophy by targeting specific hypertrophic genes. Recently published work^4^ helped to confirm this idea by identifying that all four miRNAs, when transfected, could effectively reverse hypertrophy induced by angiotensin 2 (Ang2) and sympathomimetic amines (e.g., phenylephrine and isoproterenol). More importantly, that work also showed that one of the miRNAs, miRNA106a, could be delivered to cardiomyocytes in vitro without a transfection reagent if reversibly linked to CTP, a cardiomyocyte‐specific cell penetrating peptide. It was shown that upon delivery, CTP‐miRNA106a reversed hypertrophy by downregulating HF genes CaMKIIδ and HDAC.^4^ Both genes have been shown extensively in the literature to directly induce cardiac hypertrophy when misexpressed.^6^, ^21^, ^22^, ^23^, ^24^, ^25^, ^26^, ^27^, ^28^, ^29^, ^30^

Here, we tested the ability of CTP to deliver miRNA106a to the murine heart in vivo in a fully functional form for rescuing hormonally‐induced HF (Angiotensin 2 and norepinephrine) in vitro and in vivo using an osmotic pump mouse model. We also test the ability of miRNA106a delivered by CTP to reverse predicted HF genes, including the PLCβ1 and inflammatory pathways. This is the first time CTP has been used to deliver a miRNA specifically to the heart in vivo to alter HF‐specific signalling pathways.

## 2 METHODS AND MATERIALS

### 2.1 CTP‐miRNA106a production

CTP‐miRNA106a was generated at The University of Pittsburgh's Peptide and Peptoid Synthesis Facility. The final product consists of miRNA106a conjugated to the N‐terminus of CTP (NH_2_‐APWHLSSQYSRT‐COOH) via an intervening disulfide bond (Figure S1). Production protocol has been published^4^ and is summarized in the Supporting Information Data section.

### 2.2 Animal studies

All animal protocols were approved by the Institutional Animal Care and Use Committee of Georgetown University (Protocol 2022‐0013). We generated an HF mouse model in C57BL/6 mice with continuous infusion of angiotensin‐2 (Ang2‐1.5 mg/kg/day; Sigma‐Aldrich A9525) and isoproterenol (Iso‐30 ng/kg/day; Sigma‐Aldrich 420355) using osmotic pumps (Alzet Inc.; Cat# 2006) to induce HF in mice.^31^, ^32^, ^33^, ^34^ The osmotic pump can provide continuous infusion for 6–7 weeks. A detailed protocol of the osmotic pump surgery and pump priming can be found in the Supporting Information Materials and Methods section.

Heart failure indicators were measured at the beginning, pre‐CTP‐miRNA106a Injection, and end of the experiment using a VisualSonics VEVO 3100 ultra‐high‐resolution ultrasound machine, which collected multiple heart parameters [e.g., ejection fraction (EF), left ventricle mass (LVmass), fractional shortening (FS), etc.] for each mouse. In‐depth methods are described in the Supporting Information Methods section. Post‐euthanasia hearts and multiple organs were harvested for RT‐qPCR, protein analyses (Western blots), and immunofluorescence after cryosectioning and fixation with 4% paraformaldehyde. Figure S2 illustrates the timeline for mouse experiments.

### 2.3 Testing biodistribution using Cy5.5‐CTP‐miRNA106a‐Cy3

Wild‐type CD1, 6–8‐week‐old mice (male and female, 25–35 g) were utilized for the dual‐labelled CTP‐miRNA106a biodistribution studies. CTP was synthesized with its C‐terminus labelled with Cy5.5, and conjugated at its N‐terminus via a disulfide linker to Cy3 labelled miRNA106a. Mice were injected intravenously with 10 mg/kg Cy5.5‐CTP‐miRNA106a‐Cy3 conjugated, followed by euthanasia with inhalational CO_2_ at the indicated time points (30 min, 1, 3.5, 6 and 24 h). Heart and multiple organs were harvested, placed in OCT compound, and snap frozen in liquid nitrogen for later processing and cryosectioning.

### 2.4 Cell culture

Human cardiomyocytes (hCMs) were cultured in complete growth media with serum and antibiotics (Celprogen, Inc; Cat# 36044‐15VT) in a CO_2_ water jacketed incubator with 37°C and 5% CO_2_, grown to 50% confluence, followed by daily treatments with 100 nM phenylephrine (PE, Sigma‐Aldrich 59‐42‐7) and 10 nM angiotensin 2 (Ang2, Preprotech Inc). PE is used instead of ISO because PE targets a1‐adrenergic receptors, which can promote remodelling of hCMs.^35^ It is identified in the literature that Ang2 is often paired with PE for in vitro cardiac hypertrophy experiments, while ISO is paired with Ang2 in vivo; however, both ISO and PE have been shown to induce hypertrophy and remodelling.^35^

The hCMs used were previously examined by RNA sequencing, verifying their hCM authenticity.^36^ To identify hypertrophic response, hCMS were treated with PE/Ang2 for 24, 72 and 144 h. For all experiments, ‘Prevention’ equals 72 h pretreatment with CTP‐miRNA106a, followed by 72 h of Ang2/PE for a total of 144 h. ‘Reversal’ equals 144 h of daily treatment of Ang2/PE, adding CTP‐miRNA106a at the 72 h mark (halfway through the Ang2/PE treatment). The latter approach requires CTP‐miRNA106a to counteract Ang2/PE stimulation.

### 2.5 Enzyme‐linked immunosorbent assay

A PKC kinase activity assay (Enzo Life Science Inc.; Cat# ADI‐EKS‐420A) and IL1β Human Elisa Kit (Enzo Life Science Inc.; Cat# ADI‐900‐130A) were used to determine the PKC activity change per assay protocol. Directions and analyses are explained in detail in the Supporting Information Data section.

### 2.6 RNA extraction and reverse transcription quantitative polymerase chain reaction

RT‐qPCR analyses in cells were performed following the instructions from the Power SYBR Green PCR Master Mix from Thermo Fisher Scientific (Cat# 4367659) and the iTaq universal SYBR Green Supermix from Bio‐Rad (Cat# 1725121). Primer sequences are given in Table 1.

**TABLE 1:** Primer sequences of target genes.

Target gene	Primer sequence
PLCβ1	F‐5′‐GCGCAAAGTAAACGGCAGA‐3′
	R‐5′‐ACCACTTGAGAGCTTGAGGG‐3′
Desmoplakin	F‐5′‐GGAGCGAGATCCCTCCAAAAT‐3′
	R‐5′‐ GGCTGTTGTCATACTTCTCATGG‐3′
miR106a	5’‐CAAAGTGCTAACAGTGCAGGTAG‐3’

### 2.7 Luciferase assays, fluorescence‐activated cell sorting, and immunofluorescence analyses

We followed the protocol for IF analyses found in a study.^4^ A more detailed description has been placed into the supplemental data section. Luciferase assays were conducted to identify and confirm 3′UTR targets for miRNA106a. PLCβ1 3′‐UTR luc construct (Genecoipea Inc., Cat# pEZX‐MTO6) was transfected into HEK293 cells (ATCC; CRL‐1573), followed 24 h later by transfection of miRNA106a, miRNA93 (same seed sequence as 106a), or miRNA106a with a single nucleotide mutation in the seed sequence. Results were analyzed using a luminometer. Another luciferase assay was performed after transfecting with a plasmid expressing luciferase under an NF‐κB‐binding promoter sequence into hCMs to study the effects of miR106a on NF‐κB response. The cells were collected using a dual‐luciferase reporter assay kit (Promega Inc.; Cat# Nano‐Glo Dual‐Luciferase Reporter Assay System, Cat# N1610) and transferred into a 96‐well plate detector in a Wallac VICTOR2 96‐well plate reader. Mimic miRNA106a, miRNA93, and mutated miRNA106a were purchased from Horizon‐Dharmacon Inc. (Custom miRNA design link).

Fluorescence‐activated cell sorting (FACS) was used to determine the impact of miRNA106a on the downstream factor IL‐1β. Antibody for IL‐1b FACS was purchased from Santa Cruz (Cat# 12742). Protocol details can be found in the Supporting Information Data section.

### 2.8 Calcium imaging

Cells were loaded with 1 µM Fluo‐4‐AM (Thermo Fisher Scientific; Cat# F14201) in a physiological buffer solution containing 140 mM NaCl, 4 mM KCl, 1 mM MgCl_2_, 1.2 mM CaCl_2_, 10 mM HEPES, and 5 mM glucose (pH 7.3). The cells were locally super‐fused with a buffer with or without drugs using a 250 µm cannula connected to a valve‐controlled gravity‐fed super‐perfusion system. Cells were imaged with ×20 objective using a Nikon TE2000 microscope with an excitation filter of 480 ± 15 nm and an emission filter of 535 ± 25 nm. The images were captured by a Retiga 3000 digital camera (QImaging), with analysis performed offline using ImageJ.

### 2.9 Statistical analyses

Statistical analyses are described in detail in the Supporting Information Materials section. Briefly, one‐way ANOVAs were performed on experiments where there were more than two treatments (e.g., untreated vs. Ang2/PE‐treated vs. Ang2/PE + CTP‐miRNA106a‐treated). When significance was identified, post hoc unpaired *t*‐tests were used. *N* ≥ 3 was the minimum measurement in each group; however, in many cases, *N* > 10 was used for analyses. *N*‐values can be found in the figure legends.

## 3 RESULTS

**FIGURE 1:**
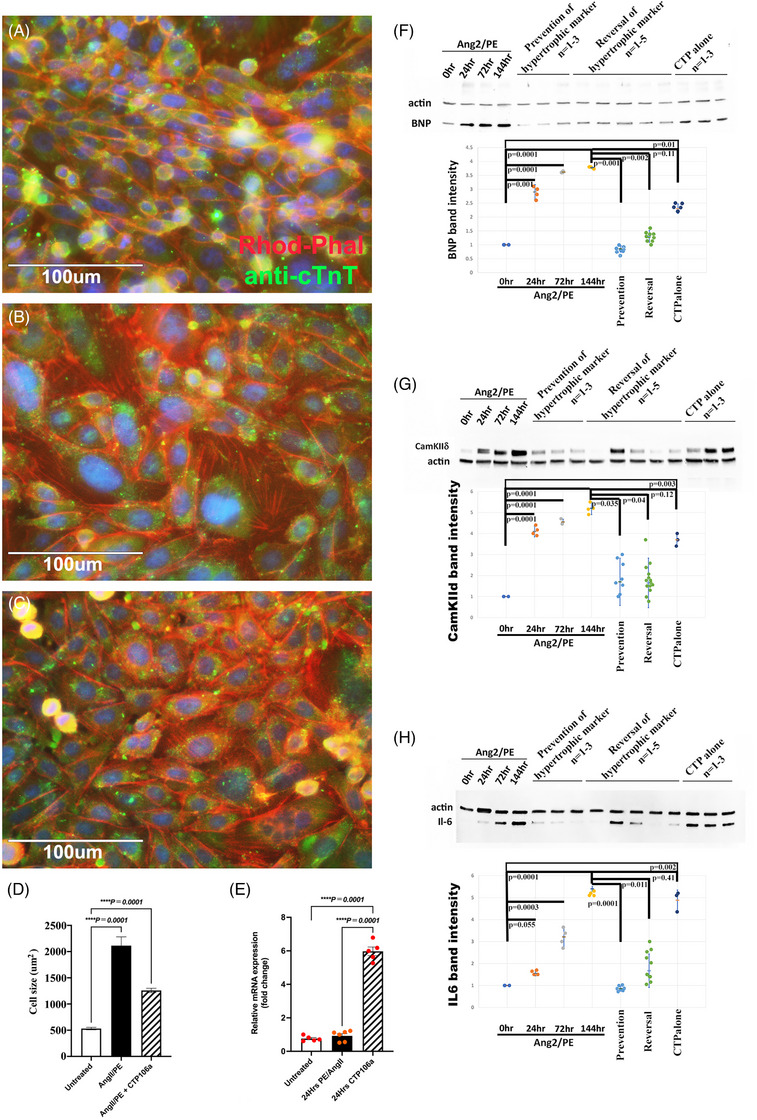
Ang2/PE‐induced hypertrophic responses in hCMs can be prevented or reversed by CTP‐miR106a. (A) hCMs were left untreated, or (B) treated with Ang2/PE for 72 h, or (C) treated with Ang2/PE for 72 h, adding CTP‐miR106a after 24 h, followed by a 48 h incubation. CTP‐miRNA106a treatment in (C) shows evidence of reversal of hypertrophy by CTP‐miRNA106. (D) Outlining each cell in (A–C) using ImageJ, the total pixel area was measured and graphed, confirming significant reversal of Ang2/PE‐induced hypertrophy by CTP‐miRNA106a. *N* = 100 cells measured for each treatment. (E) Rt‐qPCR probing for miRNA106a revealed a basal level of miRNA106a in untreated hCMs. This level was not significantly different in Ang2/PE‐treated hCMs; however, hCMs treated with CTP‐miRNA106a for 24 h resulted in significantly elevated levels of miRNA106a. (F) Western blot using the HF marker BNP shows a typical elevated response to Ang2/PE that is either prevented or reversed using CTP‐miRNA106a. CTP alone has no effect. The dot plot shows band intensities for each treatment. (G, H) The same Western pattern seen in **F** is observed for HF markers CamKIIδ and interleukin‐6 (IL‐6). Dots represent replicate quantifications for all Western blots. *N* = 4 for controls 0–144 h Ang2/PE. *N* = 9 for preventative experiments. *N* = 13 for reversal experiments. *N* = 9 for CTP‐alone experiments. Note that some dots overlap others. One‐way ANOVA identified significance, followed by unpaired *t*‐tests comparing two variables at a time to identify significance.

The results in Figure 1 confirm and expand on our prior data^4^ showing that CTP‐miRNA106a can reverse hypertrophy (Figure 1A–D). This in vitro model system comprises a hCM cell line comparing Ang2/PE‐treated to untreated controls and Ang2/PE‐treated to Ang2/PE‐treated + CTP‐miRNA106a. ImageJ analyses using pixel area of cells outlined by Rhodamine‐phalloidin (ACTB) identified hypertrophy of hCMS (Figure 1B) treated with Ang2/PE as well as reversal of hypertrophy after culture in CTP‐miRNA106a (Figure 1C,D). Rt‐qPCR of miRNA106a identified CTP as having delivered a significant amount of miRNA106a (Figure 1E). Treatment with Ang2/PE did not significantly increase endogenous levels of miRNA106a when compared with untreated hCMs (Figure 1E). Western blot analyses of three HF markers (BNP, IL‐6, and CamKIIδ)^6^, ^37^, ^38^ provided further evidence that CTP‐miRNA106a could both prevent and reverse upregulation of proteins that play pivotal roles in HF physiology (Figure 1F–H).

We then tested the biodistribution of CTP‐miRNA106a using a dual‐labelled CTP‐miRNA106a conjugate. The correct mass of the final conjugate (Figure S1A) was verified by MALDI‐TOF analysis as having a molecular weight of 10 696 (Figure S1B). To test the transfection ability of the dual‐labelled CTP‐miRNA106a, hCMs were incubated for 30 min with 500 nM of the conjugate, with results showing uptake of both miRNA106a‐Cy3 (green) and CTP‐Cy5.5 (red) (Figure S1C–E). Remarkably, after 5 h of incubation, CTP‐Cy5.5 staining dissipated, leaving only the miRNA106a‐Cy3 portion of the drug within cells (Figure S1F–H). CTP is most likely extruded from the hCMs by exocytosis, as shown previously.^39^, ^40^

**FIGURE 2:**
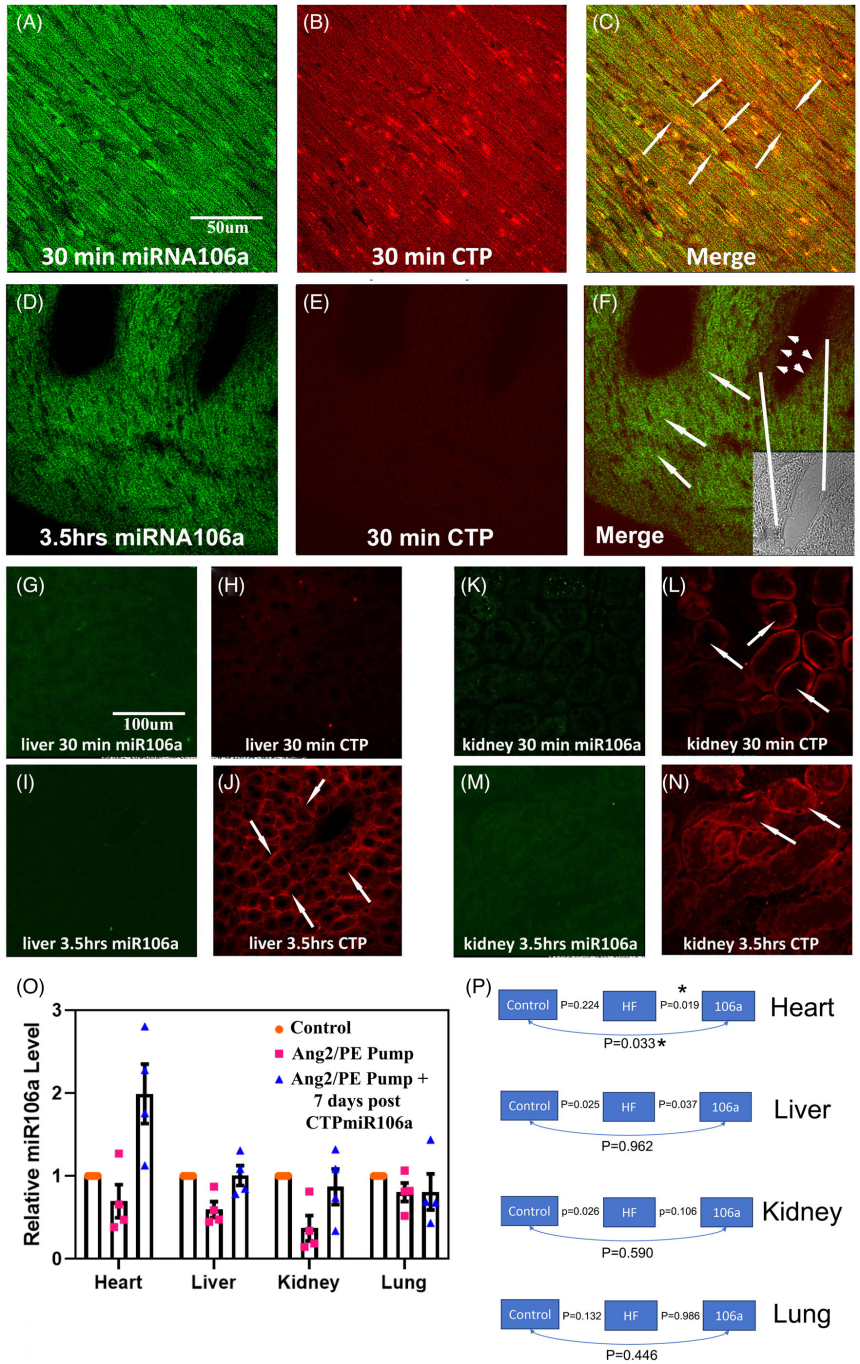
10 mg/kg dual reporter Cy3‐miRNA‐CTP‐Cy5.5 (Figure S1) was injected systemically into mice, followed by analyses of heart, liver, and kidneys 30 min and 3.5 h later. (A–C) CTP delivers miRNA within 30 min. (D–F) miRNA106a remains in the heart, while CTP dissipates from the heart and is filtered by the liver and kidneys (G–O). Arrows in (C) and (F) point to myocytes. Note: Myofibrils are visible within myocytes. Inset in (F) shows a phase contrast image of a larger blood vessel and its boundaries (white lines). Note little to no staining of the endothelial layer (arrowheads) of the blood vessel. Arrows in (J) point to CTP in liver lobules. Arrows in (L) and (O) point to CTP in nephrons. Note: no miRNA106a is observed in the liver or kidneys. (P) rt‐qPCR probing for miRNA106a provided further evidence that CTP delivers miRNA106a cargo to the heart. Mice (*N* = 4) were analyzed at the end of each experiment, which coincided with ∼day 42 or 1 week after the last of four systemic injections of CTP‐miRNA106a. (Q) Statistical analyses show that only the hearts from CTP‐miRNA106a‐injected mice showed significantly elevated miRNA106a levels (blue triangles). One‐way ANOVA identified significance, followed by unpaired t‐tests comparing two variables at a time to identify significance. Statistics for each organ are shown to the right of the graph.

Next, we analyzed the dual‐label dye in vivo by systemic injection into mice. The cardiac uptake in cardiomyocytes (note striations) of the conjugate at 30 min post‐intravenous injection reported positive for both CTP (Cy5.5) and miRNA106a (Cy3) (Figure S2; autofluorescence control Figure S1I–J). However, at 3.5 h post‐injection, only the miRNA106a‐Cy3 remained in the heart with CTP‐Cy5.5 localized primarily in the kidney and liver lobules likely slated for renal and hepatobiliary excretion, respectively. MiRNA106a‐Cy3 signal was not observed in the kidneys or liver, even at 3.5 h post‐injection. Using rt‐qPCR, we double checked the distribution of miRNA106a in vivo after intravenous injection of CTP‐miRNA106a (without fluorescent dyes; Figure 2P,Q). Expression persisted 1 week after the fourth/last injection of CTP‐miRNA106, where miRNA106a was significantly elevated only in the hearts of mice injected with CTP‐miRNA106a. Figure 2Q provides the statistical analyses for the qPCR data graphed in Figure 2P. The data show that CTP did not deliver miRNA106a to the lung, liver, or kidneys. It is important to note that CTP‐miRNA106a was delivered to the heart within 30 min after tail vein injection (Figure S1K). Interestingly, the liver and kidney showed significantly less expression of miRNA106a in mice containing Ang2/Iso pumps + saline injections (heart failure mice). We do not know the cause of this lowered expression; however, we believe we are the first group to report this finding. The subsequent rise in miRNA106a after injection of CTP‐miRNA106a in these organs (third bar for each organ in the graph), we surmise, is due to acclimation to Ang2/Iso and not CTP delivering miRNA106a to those organs because none of the organs show Cy3‐miRNA106a staining. Collectively, the evidence demonstrates that CTP delivers miRNA106a specifically to the heart within 30 min and that significant miRNA106a expression in the heart lasts for at least 1 week postinjection.

**FIGURE 3:**
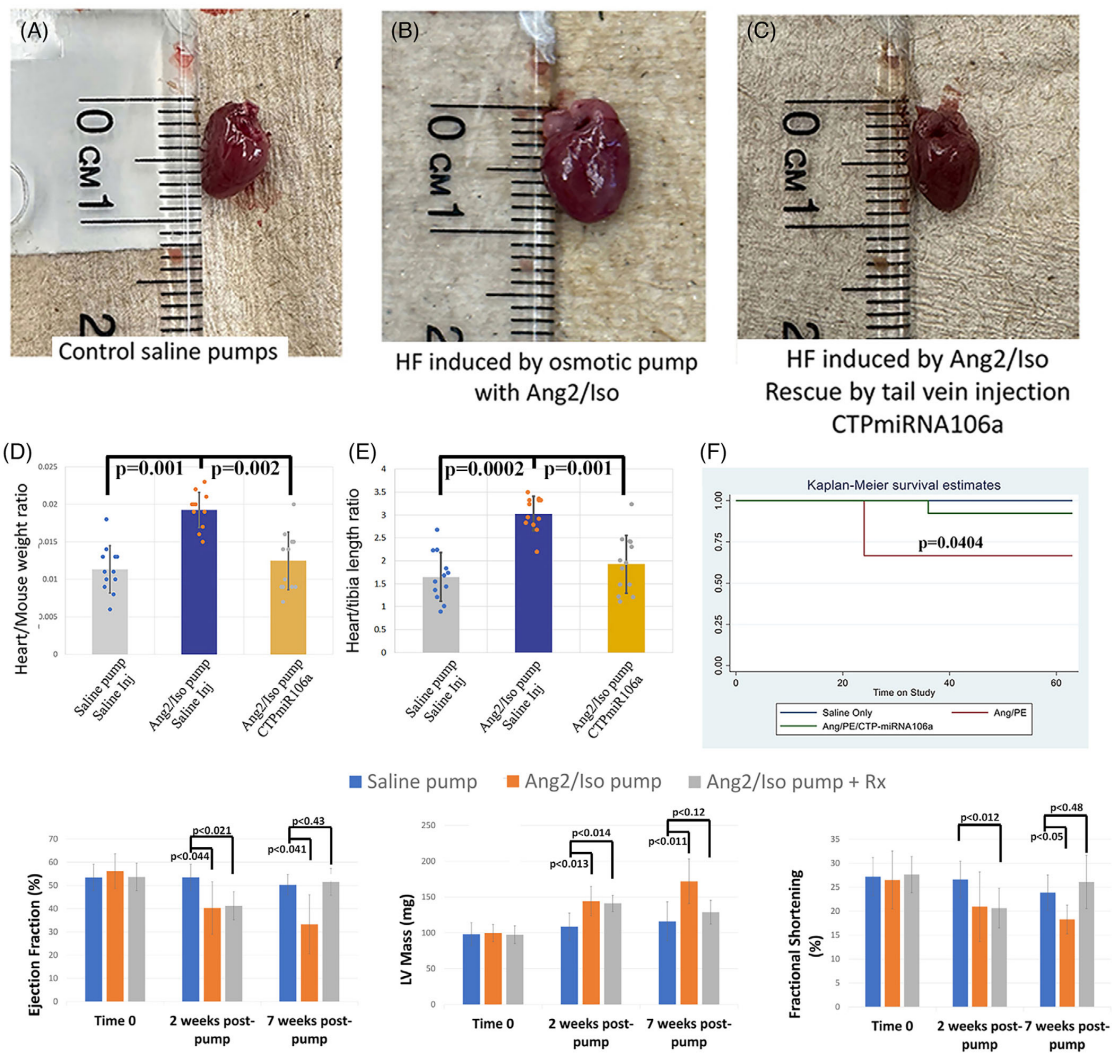
Morphological and functional analyses of hearts from control mice (saline injected) compared with mice treated with 1.5 mg/kg/day Ang2/Iso for 6 weeks + saline, and mice treated with Ang2/Iso and at least 4 weekly systemic injections of CTP‐miRNA106a. (A–C) Hearts from untreated (A), Ang2/Iso treated + saline injections (B), or Ang2/Iso pump + CTPmiRNA106a‐treated mice (C), show clear induction of hypertrophy with Ang2/Iso treatment (B), which was reversed by CTP‐miRNA106a treatment. Heart to mouse weight ratios (D) and heart weight to tibia length ratios (E) provided further evidence that CTP‐miRNA106a reversed cardiac hypertrophy (*N* = 11 samples each treatment). (F) The Kaplan–Meier survival curve showed CTP‐miRNA106a having a mortality benefit in Ang2/Iso treated mice. (G–I) Ejection fraction (EF), LV mass, and fractional shortening (FS) were measured for control mice treated with saline (blue bars), mice treated with Ang2/Iso/saline (orange bars), and mice treated with Ang2/Iso+CTP‐miRNA106a (grey bars). Measurements were taken at the start of the study (time 0), 2 weeks post‐Ang2/Iso treatment, and prior to euthanasia at 7 weeks (see also Figure S2). Graphs show the development of heart failure at week 2 with significant rescue of all tested parameters after a 4‐week treatment with CTP‐miRNA106a. Saline pump *N* = 15, Ang2/Iso pump *N* = 15 (two died from heart failure between week 2 and 7), Ang2/Iso pump + CTP‐miRNA106a *N* = 20 (with one death). One‐way ANOVA identified significance, followed by unpaired *t*‐tests comparing two variables at a time to identify significance.

We then commenced with experiments to test the efficacy of CTP‐miRNA106a for reversing HF morphology, molecular signatures, and physiology in vivo. To begin, five cohorts of 12 mice each were analyzed over an ∼8‐month period (illustrated in Figure S2). Figure 3 and Figure S3 show the changes in LVmass and multiple echocardiographic parameters for the various cohorts. After weekly 10 mg/kg CTP‐miRNA106a injections for four weeks, Ang2/Iso‐induced hypertrophy was reversed in all but one mouse, which died in the hand of the technician as it was being injected into the tail vein with CTP‐miRNA106a (Figure 3A–E; Figure S3). Average EF and FS were also significantly increased by CTP‐miRNA106a (Figure 3G–I). Figure 3D,E verified Heart/mouse weight ratios and Heart /Tibia length ratios in a separate set of mice containing Ang2/Iso pumps. In addition to rescuing HF parameters, mice receiving CTP‐miRNA106a injections had a significant survival advantage over untreated mice (Figure 3F). The one mouse that died in the treatment group immediately after receiving a single injection of CTP‐miRNA106a was attributed likely as an anaesthesia‐related adverse event after necropsy showed no apparent deterioration or injury to any organ. Nineteen other mice receiving weekly injections of 10 mg/kg CTP‐miRNA106a over four weeks all survived until the end of the experiment.

**FIGURE 4:**
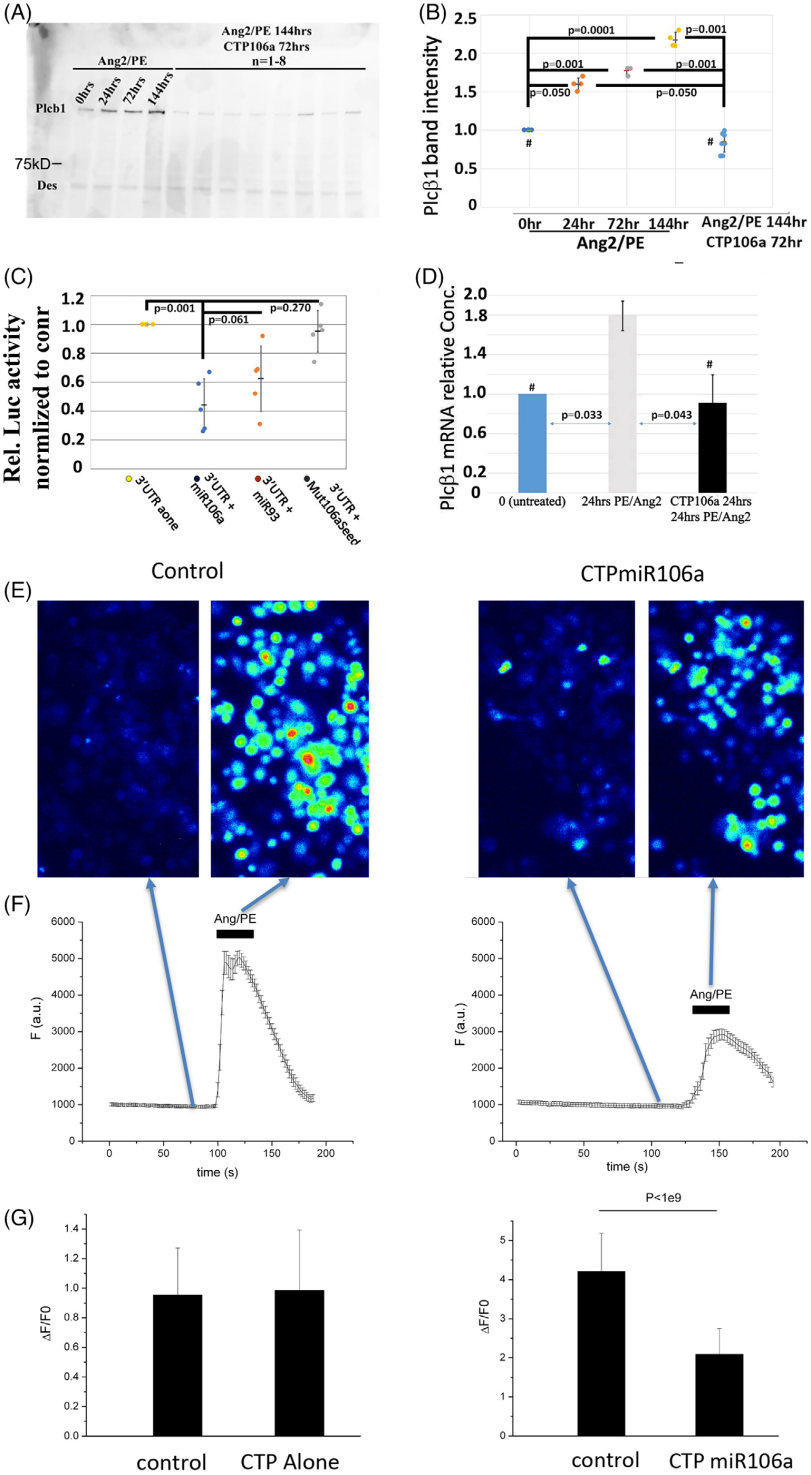
CTP‐miRNA106a targets PLCβ1, resulting in down‐regulation of PKC and IP3. (A, B) hCMs incubated in Ang2/PE over time result in increased expression of PLCβ1. In addition, 72 h of CTP‐miRNA106a after 72 h of Ang2/PE treatment results in down‐regulation of PLCβ1 to 0 h (untreated) levels. Anti‐desmin was used as a loading control. *N* = 3 for 0–144 timepoints and *N* = 24 for CTP‐miRNA‐rescued samples. (C) Confirmation that PLCβ1 is a bona fide target of miRNA106a, 3′UTRLuc assay transfected into HEK293 cells, followed 24 h later by transfection of miRNA106a, miRNA93 (same seed sequence), or miRNA106a with a single nucleotide mutation in the seed sequence. miRNA106a significantly decreased luciferase activity by ∼60% confirming MiRNA106a targets PLCβ1. MiRNA93 also decreased luciferase activity, but only by ∼40% (miR93 has the same seed sequence as 106a but a different non‐seed sequence). A mutated miRNA106a had no effect. (D) Supporting the Western analyses, rt‐qPCR showed a significant increase in PLCβ1 mRNA after 24 h of Ang2/PE treatment; however, PLCβ1 mRNA was significantly decreased after the addition of CTP‐miRNA106a. These data suggest that miRNA suppresses translation by degrading PLCβ1 mRNA. (E) Pseudo‐colour images showing Fluo4 fluorescence in control (left panels) and miRNA‐treated (right panels) cells before and after application of Ang2/PE. These data show that CTP‐miRNA106a prevents Ca2+ release into the cytoplasm of hCMs. (F) (Lower graph) Mean fluorescence (±SEM) versus time for control (*n* = 19) and miRNA (*n* = 13) treated cells. (G) Mean change (±SD) in Fluo4 fluorescence (ΔF/F0) for control (*n* = 18) versus CTP alone (*n* = 18) and control (*n* = 21) versus miRNA/CTP (*n* = 20). Statistical analysis for E–G is a two‐tailed *T*‐test, *p* < 1e9. Dots on graphs represent replicates (*N*). Statistical analysis for (A–D) was obtained by one‐way ANOVA, followed by unpaired *t*‐tests comparing two variables to identify significance.

hCMs were then utilized to identify CTP‐miRNA106a HF targets. Western blot and luciferase expression analyses verified in silico observations that PLCβ1 was a target for miRNA106a (Figure 4A–D). When combined, these data provided four important pieces of information: (1) Persistent Ang2/PE incubation causes increased expression of PLCβ1 over time (Figure 4A,B), (2) Luciferase assays confirmed PLCβ1‐3′UTR is targeted by miRNA106a (Figure 4A,C), (3) rt‐qPCR analyses showed that PLCβ1 gene expression increases over time in response to Ang2/PE, and (4) treating hCMs with CTP‐miRNA106a results in degradation of PLCβ1 mRNA (Figure 4D). In summary, CTP‐miRNA106a was shown to suppress translation of PLCβ1 by targeting its 3′UTR, resulting in mRNA degradation and decreased protein expression.

PLCβ1 functions by cleaving PIP2 into the bioactive lipids IP3 and DAG. One consequence of CTP delivery of miRNA106a to Ang2/PE‐treated hypertrophic hCMs should be a significant decrease in Ca^2+^ release due to decreased PLCβ1 expression. To test this hypothesis, fluorescent Ca^2+^ imaging was utilized to follow Ca^2+^ fluxes in untreated, Ang2/PE‐treated, and CTP‐miR106a‐primed hCMs that were then treated with Ang2/PE. The data showed that the Ca^2+^ flux significantly increased in hCMs after an Ang2/PE pulse; however, the Ang2/PE‐induced Ca^2+^ signal was significantly suppressed if hCMs were preloaded for 48 h with CTP‐miR106a (Figure 4E–G). Preloading with CTP alone (i.e., no miRNA106a attached) had no effect on Ang2/PE‐induced Ca^2+^ increase (Figure 4G), further confirming that the reduction in calcium flux was mediated by miRNA106a targeting of PLCβ1, resulting in decreased enzymatic effect on PIP2 (data not shown).

In vitro stimulation by continual Ang2/PE added daily to media leads to persistent PLCβ1 activation, which, in turn, leads to increased and persistent IP3/DAG production and PKC activation. Incubating hCMs in either PMA (synthetic activator of PKC) or Ang2/PE resulted in translocation of PKCα to the plasma membrane (a hallmark of PKC activation) within hCMs (Figure S4; quantified in Table 2). However, treatment with CTP‐miRNA106a prevented this translocation.

**TABLE 2:** HCM analyses of PKC localized to plasma membranes and HCM analyses of phosphorylated Cnx43.

Membrane‐bound PKCa	Untreated	30 min PMA	3 h Ang2/PE	48 h CTP‐miR106a + 3 h Ang2/PE
Avg % of cells + SD *N* = 300 cells	25 ± 5	79.3 ± 4.0 (*p* < .001)*	75.7 ± 6.0 (*p* < .0001)*	19 ± 3.6 (*p* < .37)
Phosphorylated Cnx43 (cell junctions)	Untreated	30 min PMA	72 h Ang2/PE	72 h Ang2/PE +48 h CTP‐miR106a
Avg % of cells ± SD *N* = 300 cells	4.3 ± 1.0	61 ± 5.3 (*p* < .0001)*	49.3 ± 4.2 (*p* < .0001)*	7.7 ± 2.5 (*p* < .4)

* Significant compared with untreated.

**TABLE 3:** miRNA106a predicted targets.

*Confirmed targets	3′UTR seed sequence location (blue) From miRDB.org
*CamK2δ [4]	**GenBank Accession** NM_001321589 691 **5′**ttaat **cacttta3’** 961 **5′**a **gcacttt** tgat**3’**
PLCβ1‐this manuscript	2241 **5′** tactttt **gcacttt** **3’**
*STAT3	**GenBank Accession** NM_213662 251 **5′**g **gcacttt** taaaa**3’** 541 **5′**t **gcacttt** tt**3’** 711 **5′**atccca **gcacttt** gg**3’**
CREB (cAMP pathway)	**GenBank Accession** NM_004379 1531 **5′**gtaatccca **gcacttt** gg**3’**
MAPK1	**GenBank Accession** NM_002745 411 **5′**tct **cacttta** tga**3’** 441 **5′**tagg **gcacttta** ag**3’**
MAPK4	**GenBank Accession** NM_001292040 1511 5′cctat **gcact tt** cc**3’**
MAPK8	**GenBank Accession** NM_001278548 2081 **5′**tcactgtt **gcacttt** ca**3’**
MAPK9	**GenBank Accession** NM_139068 491 **5′**a **gcacttt** ggaaa**3’**

* Significant compared with untreated.

**FIGURE 5:**
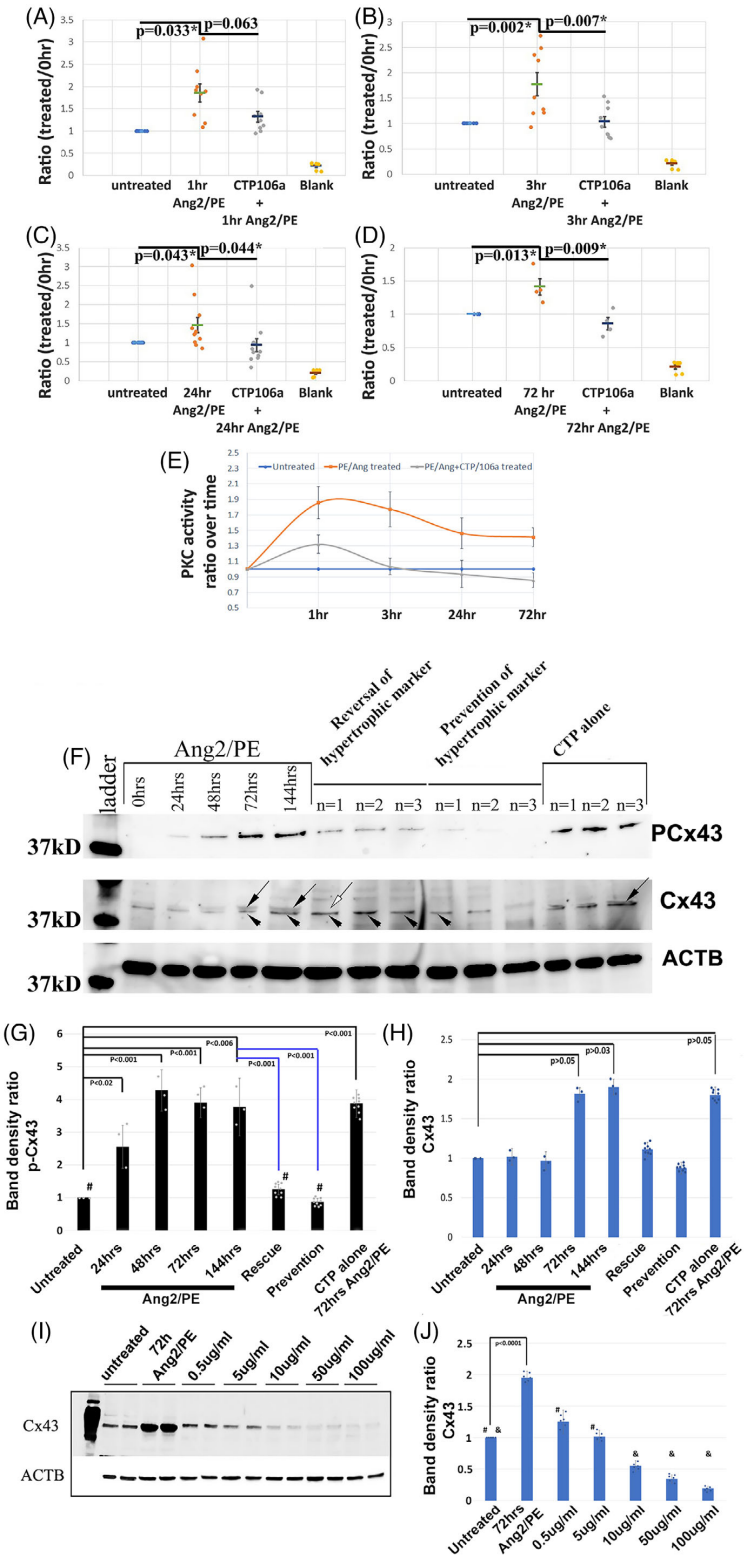
Ang2/PE activates PKC, which is subsequently suppressed by CTP‐miR106a. (A) Normalizing PKC activity ratios to untreated hCMs (0), 1 h treatment with Ang2/PE resulted in a significant elevation of PKC activity. This elevated activity could be prevented by 48 h incubation in CTP‐miR106a. (B–D) This same pattern persisted through 3 days. Asterisks (*) show significant differences. (E) Using a smooth curved graph, it became apparent that the elevated PKC activity caused by Ang2/PE was decreased in response to CTP‐miR106a. A spike in activity is observed at 1 h of Ang2/PE, followed by a decrease and eventual plateau. Incubating hCMs in CTP‐miR106a for 48 h significantly suppresses this spike and plateau of PKC activity. Each dot represents an experimental replicate (*N*). (F) Western blot analyses of pCx43 and total Cx43 confirm immunofluorescence data. Top Western blot‐Ang2/PE induces phosphorylation of Cx43 on Ser368, the PKCα phosphorylation site, over time. CTP‐miRNA106a significantly decreased phosphorylation both before (preventative approach) or after (rescue approach) incubation in Ang2/PE. Middle Western blot: Total Cx43 revealed an increase in Cx43 protein expression in response to Ang2/PE that was subtly inhibited by CTP‐miRNA106a. Bottom Western blot: antibodies to beta actin showed loading was similar in each lane. *N* = 3 for 0–144 timepoints. *N* = 9 for both Cx43 and p‐Cx43. (G) Pixel intensity ratios measured using the ImageJ histogram tool show significant increases in phosphorylation at each timepoint of Ang2/PE incubation. CTP‐miRNA106a significantly decreased phosphorylation both before and after incubation in Ang2/PE. CTP alone had no effect. # = no significant difference between CTP‐miR106a and untreated cells, supporting the idea that CTP‐miR106a can prevent and/or rescue PLCβ1/PKC over‐expression in Ang2/PE‐treated hCMs. (H) Analysis of total Cx43 showed a significant increase in protein expression at 72 and 144 h post‐Ang2/PE incubation. A marked but insignificant decrease in Cx43 expression was observed if CTP‐miRNA106a was administered before Ang2/PE treatment; however, if CTP‐miRNA106s was administered prior to Ang2/PE treatment, Cx43 expression was significantly decreased. CTP alone had no effect. Black arrows point to the top band of a doublet that increases in intensity at 48–144 h of Ang2/PE treatment. The white arrow points to the loss of the doublet when incubated in CTP‐miRNA106a. We surmise that the top band is the phosphorylated form of Cx43. (I) The algorithm at miRDB.org predicts Cx43 as a target of miRNA106a. A dose‐dependency experiment resulted in protein expression data showing that at the concentration used for knocking down HF genes (5 µg/mL, e.g., CamkIIδ does not significantly suppress Cx43 expression; however, at 2× concentration and above, significance is observed as shown in (J). # represents no significance measured using an unpaired *t*‐test compared with untreated hCMs. The & represents significance (*p* < .05 or lower) compared with untreated hCMs. J *N* = 6 experimental replications for each experimental repeat. Two separate repeats are shown on each Western blot. One‐way ANOVA identified significance, followed by unpaired *t*‐tests comparing two variables at a time to identify significance.

To concurrently test that Ang2/PE increased PKC activity and that this increase in activity could be prevented/inhibited by CTP‐miRNA106a, we utilized a PKC‐specific activity assay (Figure 5). A significant upregulation of PKC activity was observed in cardiomyocytes after 1 h (Figure 5A), 3 h (Figure 5B), 24 h (Figure 5C), and prolonged 72 h incubation in Ang2/PE (Figure 5; Table 2); however, CTP‐miRNA106a significantly prevented this Ang2/PE‐induced PKC activity. Interestingly, when viewing the data over time, PKC activity was consistently found to surge at 1 h post‐Ang2/PE incubation. This surge was followed by a small reduction in activity that plateaued at the 72 h time‐point (Figure 5E). Based on these results, the evidence highly suggests that CTP‐miRNA106a can suppress PKC activity induced by sustained Ang2/PE stimulation.

A significant body of research has revealed that phosphorylation of the gap junction protein, Cx43, at serine 368 is mediated by PKCα. This specific phosphorylation event leads to decreased gap junctional conductance.^41^, ^42^ Consequently, we utilized Cx43 as an assay for PLCβ/PKC activity and as a surrogate marker for reversal of cellular HF phenotype. Immunofluorescence analyses of Cx43 in untreated hCMs displayed a few positive regions of phosphorylation (arrows Figure S5A), while hCMs incubated in the PKC activator, PMA, resulted in a clear, increased phosphorylation pattern of Cx43 between cells within 3 h (Figure S5B). hCMs treated with Ang2/PE for 24 h of Ang2/PE also showed cell membrane localization for phosphorylated Cx43 (pCx43: Figure S5C); however, this phosphorylation event was reversed in hCMs treated with Ang2/PE for 72 h, followed by addition of CTP‐miRNA106a for 48 h (Figure S5D). Quantification of each experiment identified upregulation of pCx43 in Ang2/PE‐treated cells versus untreated cells, with CTP‐miR106a significantly inhibiting this upregulation (Table 2). These data suggest miRNA106a delivered by CTP can reverse Cx43 hyperphosphorylation.

Western blot analyses confirmed immunofluorescence results (Figure 5F–H). An interesting observation in the Western blot images of total Cx43 was the development of doublet bands of Cx43 after 48 h+ of treatment with Ang2/PE (Figure 5F). The top band of the doublet fell below the level of detection in hCMs incubated in CTP‐miRNA106a; however, the doublet returned in hCMs treated with CTP alone (no miRNA106a attached) and incubated in Ang2/PE. The data suggest that the top band could be the phosphorylated form of Cx43 and that CTP alone had minimal effect on phosphorylation of Cx43 in hCMs treated with Ang2/PE. Another observation was a measurable increase in total Cx43 in response to Ang2/PE that trended downward in hCMs treated with CTP‐miRNA106a for at least 24 h, followed by treatment with Ang2/PE (Figure 5H). A search on the miRDB.org website identified Cx43 as a potential target for miRNA106a. Although we saw a marked decrease in Cx43 in hCMs treated with CTP‐miRNA106a, the decrease was subtle compared with the decrease in phosphorylation. We hypothesized that the concentration of CTP‐miRNA106a was not sufficient to significantly knockdown Cx43. To test this hypothesis, we incubated hCMs in CTP‐miRNA106a (no Ang2/PE treatment) for 72 h at stepwise increasing concentrations. Western blot analyses showed that significant downregulation of Cx43 was observed at 10 µg/ml CTP‐miRNA106a, 2× the amount we used for experiments in each previous figure (Figure 5I,J). The evidence suggests that Cx43 is a target of CTP‐miRNA106a, but at concentrations significantly higher than what is needed to reverse the underpinnings of HF.

**FIGURE 6:**
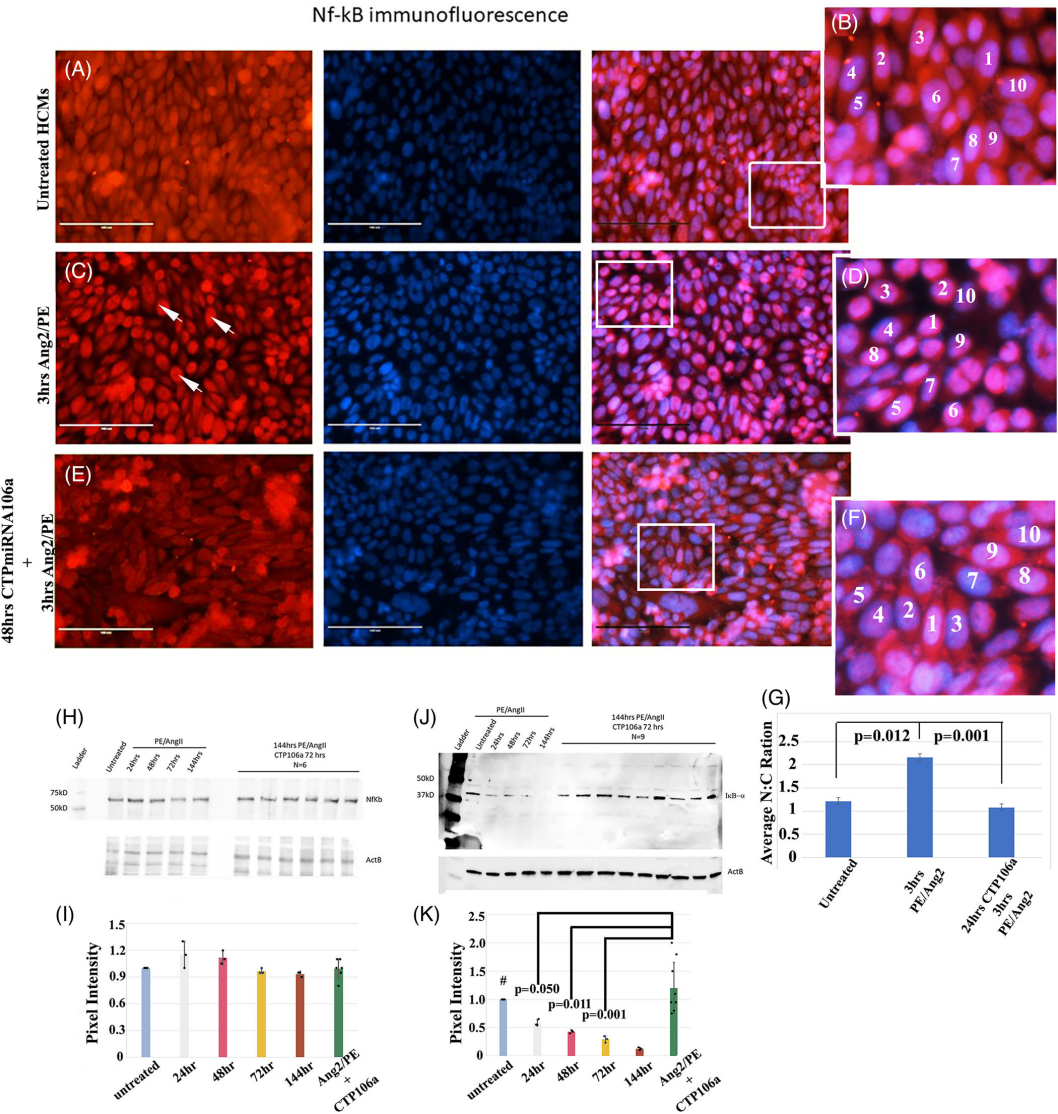
Anti‐NF‐κB antibodies/594 secondary abs show that the translocation of NF‐κB into the nucleus induced by Ang2/PE is inhibited by CTP‐miRNA106a. (A row) NF‐κB in untreated hCMs shows a diffuse NF‐κB signal. DAPI staining of nuclei in all middle images. Merged image in all the right images. (B row) 3 h of Ang2/PE results in translocation of NF‐κB into the nucleus (arrowheads). (C row) Incubating hCMs in CTP‐miRNA106a for 48 h prior to 3 h Ang2/PE treatment shows a diffuse NF‐κB pattern similar to that of untreated. White boxes identify blown‐up areas. (G) Quantification of red NF‐κB signal intensity (Numbered nuclei) using Image J in cytoplasm versus nucleus shows a significant increase of nucleus:cytoplasm ratio in Ang2/PE‐treated hCMs that is reversed in CTP‐miRNA106a pre‐treated hCMs. (H, I) Western blot and quantification of *N* = 3 blots (I) shows no significant change in expression of P65/NF‐κB in treated cells; however, (J) and (K) show that the IκΒ−α degradation induced by Ang2/PE is significantly inhibited by CTP‐miRNA106a. *N* = 3 for 0–144. *N* = 18 for both NF‐kB and Ikba Western CTP‐miRNA106a‐rescued samples. One‐way ANOVA identified significance, followed by unpaired t‐tests comparing two variables at a time to identify significance.

While our in vivo model system using Ang2/Iso osmotic pumps activates pathways that induce HFrEF, we wanted to ascertain if CTP‐miRNA106a could possibly affect other pathways involved in HF, such as the inflammation arm of HF. One of the main signalling pathways implicated in inflammation is the NF‐κB pathway. Immunofluorescent staining was utilized to compare untreated hCMs to hCM treated for 3 h with Ang2/PE or hCMs treated with CTP‐miRNA106a, followed by 3 h Ang2/PE. We used a 3 h incubation here (instead of 24 h or more) because we wanted to catch any initial spike in NF‐κB translocation to the nucleus. Three hours of Ang2/PE clearly and quantifiably caused NF‐κB to enter the nucleus in most hCMs; however, this was prevented in cells treated with CTP‐miRNA106a for 48 h followed by 3 h of Ang2/PE (Figure 6A–F). The nuclear/cytoplasmic signal ratio of NF‐κB confirmed that CTP‐miRNA106a inhibited nuclear localization caused by Ang2/PE (Figure 6G).

Because we only observed a shift in NF‐κB localization in response to Ang2/PE after a short 3 h incubation, we extended the time variable of Ang2/PE incubation for Western blot analyses in order to identify any increase or decrease in NF‐κB expression over time. Interestingly, analyses of NF‐κB (p65 subunit) in untreated versus Ang2/PE‐treated hCMs showed only a trending loss of NF‐κB over time (Figure 6H,I). However, testing for the NF‐κB inhibitory protein nuclear factor of kappa light polypeptide gene enhancer in B‐cells inhibitor, alpha (IκBα), we found a significant decrease in its expression starting 24 h after treating with Ang2/PE. More importantly, we found that CTP‐miRNA106a reversed the loss of IκBα caused by Ang2/PE (Figure 6J,K). Note that while the immunofluorescence study in (Figure 6A,F) was structured to identify if CTP‐miRNA106a prevented an increase/decrease in NF‐κB and/or prevented NF‐κB entry into the nucleus induced by Ang2/PE, the Western analyses were structured to identify if CTP‐miRNA106a could ‘reverse’ the actions of Ang2/PE‐induced NF‐κB activation. The evidence supports that CTP‐miRNA106a reverses NF‐κB activity by rescuing IκBα expression.

**FIGURE 7:**
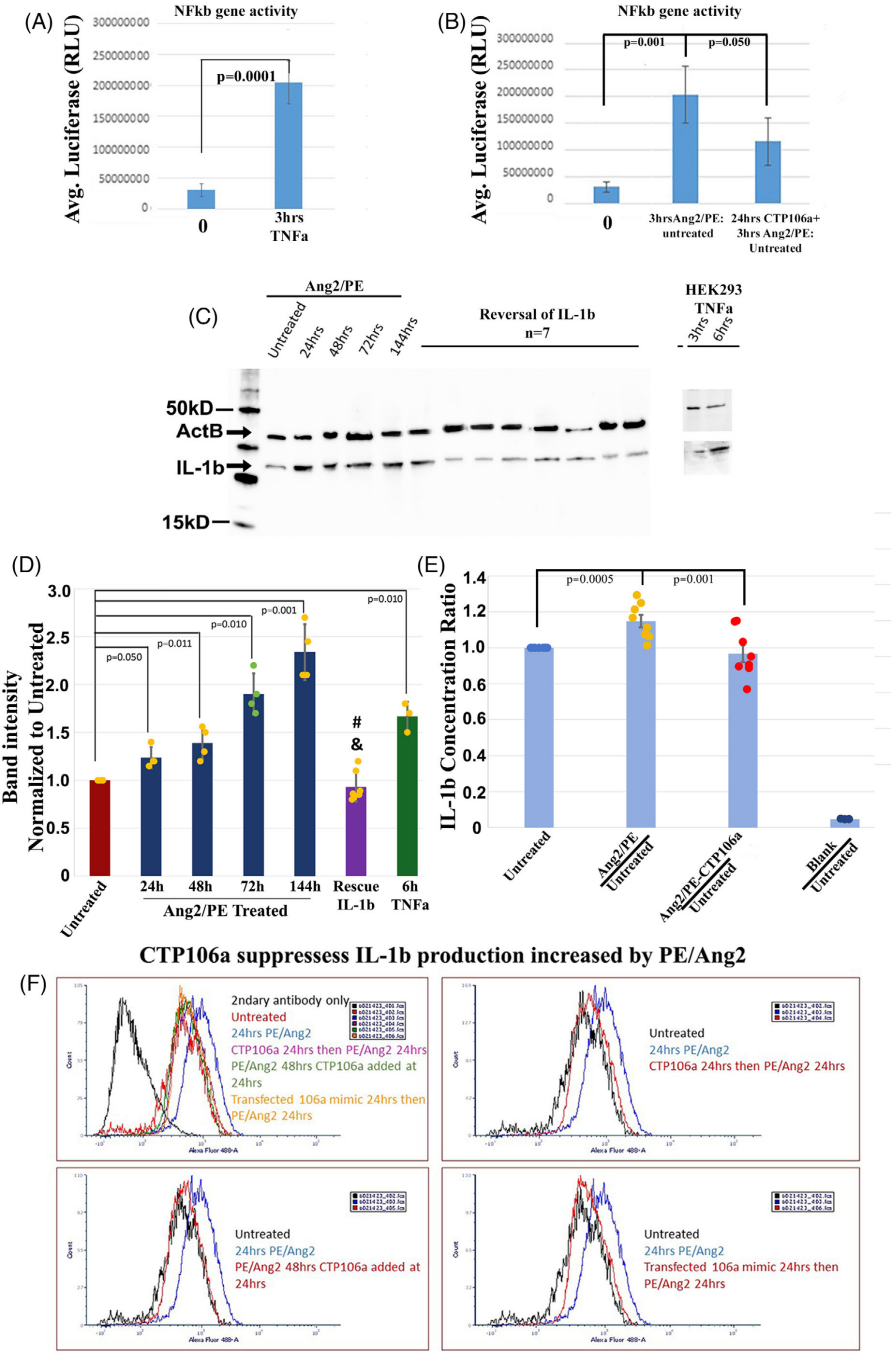
The NF‐κB response reporter gene (Promega's pNL3.2.NF‐kB NlucP/Nf‐kB/hygro) was transfected into hCMs to identify Nfκβ gene activity. (A) As a control, Tnfa was added to hCMs, resulting in a significant elevation in luciferase activity. (B) Incubating hCMs in Ang2/PE for 3 h resulted in a significant increase in NF‐κB response. This increase was prevented if hCMs were subjected to CTP‐miRNA106a prior to the addition of Ang2/PE. C‐D) One downstream gene activated by NF‐κB is IL‐1b. Western analyses showed significant increases in IL‐1b beginning at 24 h through 144 h of Ang2/PE incubation. Here, CTP‐miRNA106a was added to hCMs 72 h after Ang2/PE incubation (Rescue), resulting in a significant reduction in IL‐1b protein expression *N* = 21 (#*p* < .001 for all timepoints except untreated. & = No significant difference compared with untreated). Protein expression in CTP‐miRNA106a‐treated cells was equivalent to untreated hCMs (i.e., rescued). (E) The Media from hCMs treated for 24 h with Ang2/PE and hCMs pre‐treated with CTP‐miRNA106a, followed by Ang2/PE for 24 h, were compared with media from untreated hCMs using a sensitive ELISA assay. The secretion of IL‐1b produced in hCMs by Ang2/PE was suppressed in CTP‐miRNA106a + Ang2/PE‐treated hCMs. The blank is to show no background from the plate and media. (F) FACs’ analyses of hCMs treated with brefeldin A for the last 6 h of a 24 h Ang2/PE incubation further confirmed the increased expression of IL‐1b (blue bell curve). The upper right panel shows a clear shift of cells (red curve) back towards untreated levels if incubated in CTP‐miRNA106a prior to Ang2/PE. The lower left panel shows the same shift of hCMs (red curve) back towards baseline if hCMs are treated with Ang2/PE first for 24 h, followed by the addition of CTP‐miRNA106a for at least 24 h. As a control, miRNA106a (no CTP) was transfected into hCMs using a lipid‐based transfection reagent, followed by Ang2/PE. The results show that the shift in cells (red curve) back to baseline was caused by miRNA106a. *N* = 3 for Western 0–144 timepoints. *N* = 21 for CTP‐miRNA106a rescued samples. One‐way ANOVA identified significance, followed by unpaired t‐tests comparing two variables at a time to identify significance.

To verify our Western blot results, hCMs were transfected with an Nfκβ response element expressing luciferase when NF‐κB is bound. Serving as a control, hCMs were treated for 3 h with TNFα, a known agonist of the NF‐κB pathway^43^ (Figure 7A). TNFα significantly increased luciferase activity, demonstrating the activity of the response element in hCMs. Interestingly, treating hCMs with Ang2/PE for 3 h resulted in a significant increase in NF‐κB activity; however, pretreating hCMs with CTP‐miRNA106a for 48 h, followed by Ang2/PE, resulted in a significant decrease (prevention) in NF‐κB activity (Figure 7B). We then analyzed interleukin‐1β (IL‐1β), a pro‐inflammatory cytokine directly downstream of NF‐κB activation. Western blot analyses demonstrated that Ang2/PE treatment caused a consistent increase in IL‐1β expression over time; however, adding CTP‐miR106a at the 72 h mark of the 144 h Ang2/PE treatment resulted in IL‐1β returning to levels observed in untreated hCMs (Figure 7C,D). We then used ELISA analyses to test for secreted IL‐1β in the media used to grow hCMs. Media from hCMs treated with Ang2/PE for 24 h resulted in a significant increase in IL‐1β secretion. Interestingly, IL‐1β secretion induced by Ang2/PE could be significantly prevented by CTP‐miRNA106a (Figure 7E). Finally, using FACS analyses of hCMs, we further identified that CTP‐miRNA106a could both ‘prevent’ and ‘reverse’ production of IL‐1β (Figure 7F). Treating hCMs with Ang2/PE for 24 h (with a 1000× dilution of brefeldin A solution for the last 6 h), IL‐1β levels were markedly higher (blue bell curve line). CTP‐miRNA106a pretreatment before Ang2/PE or post‐treatment after Ang2/PE both resulted in cells with down‐regulated levels of IL‐1β. A control experiment where miRNA106a was transfected (no CTP linkage) using Mirius Transit 2 transfection reagent also demonstrated a decrease in IL‐1β expression, providing evidence that the suppression was due to miRNA106a and not the CTP (Figure 7F). When taken together, these data provide strong evidence that in hCMs, activation of the NF‐κB pathway leads to production of proinflammatory cytokines that can be prevented or reversed/suppressed by CTP‐miRNA106a.

**FIGURE 8:**
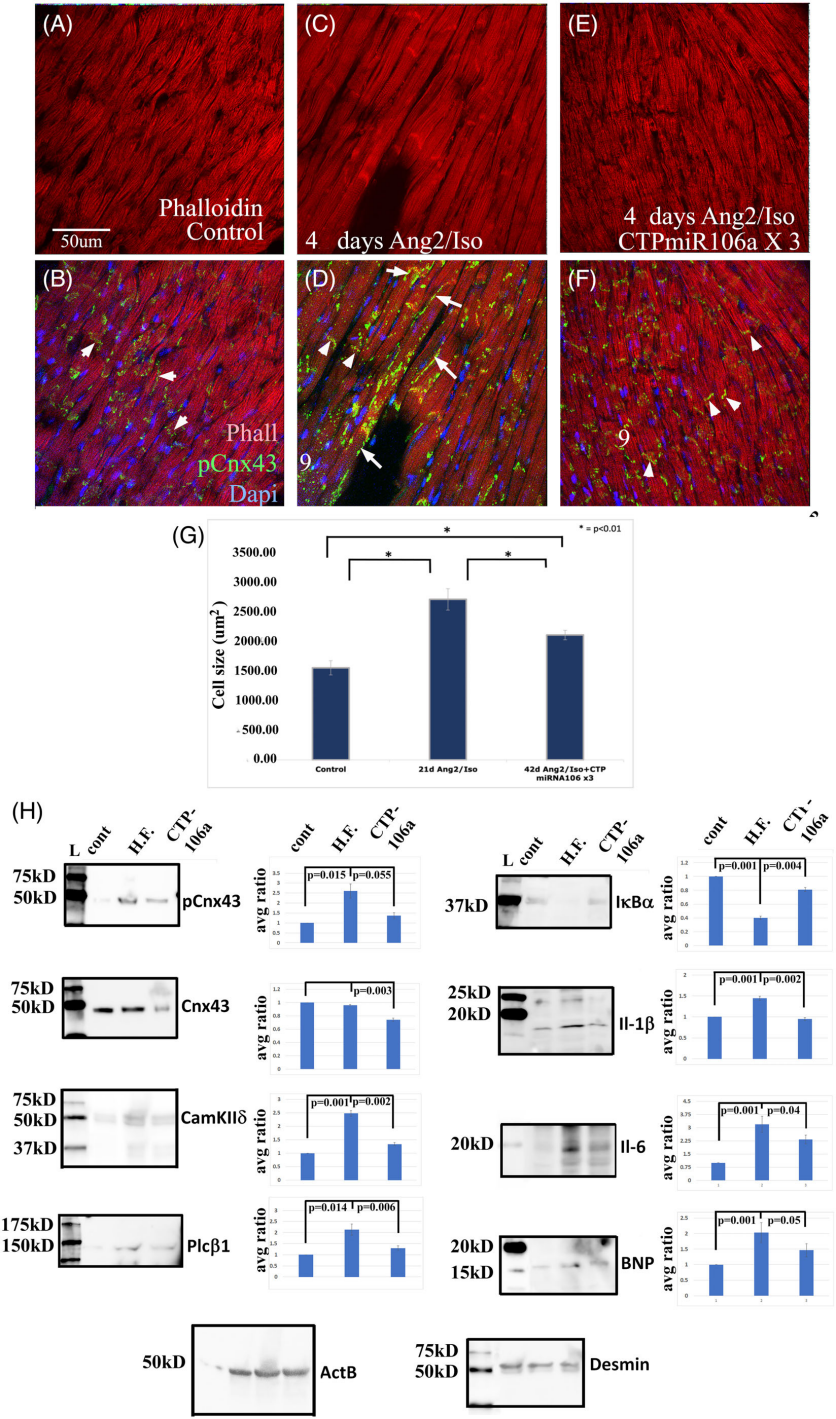
Heart tissue from mice shows that CTP‐miRNA106a rescues hypertrophic disease. (A, B) Heart tissue from control mice with saline‐filled osmotic pumps + saline injection identified a baseline level of pCx43 at intercalated disks (arrowheads). (C–D) Heart tissue from a mouse containing an osmotic pump filled with Ang2/Iso + saline injections (HF model) clearly showed an increased level of pCx43 within intercalated disks (arrowheads) and at sites located on the lateral sides of cells (arrows). (E–F) Heart tissue from mice containing a pump filled with Ang2/Iso + 4 weekly injections of CTP‐miRNA106a resulted in levels of pCx43 at intercalated disks (arrowheads) similar to untreated controls (A, B) and virtually no lateral PCx43 staining. Note the markedly larger hypertrophic cells in C and D compared with (A, B) and (E, F). (G) Quantification of cells in (A, F) shows the onset of hypertrophy in Ang2/Iso pump‐containing mice and its reversal after injection with CTP‐miRNA106a. (H) Western Blots for each protein observed in our in vitro experiments replicated the rescue by CTP‐miRNA106a. Quantification of band intensity is represented by the graphs immediately to the right of each blot. The overall results show that CTP‐miRNA106a rescues each marker of HF caused by Ang2/Iso pump secretion. Cont. = control saline pump + saline injection, HF = Ang2/Iso pump + 4 weekly saline injections, CTP‐miRNA106a = Ang2/Iso pump + 4 weekly CTPmiRNA106a injections. *N* = 3 sets of mice for each treatment. ActB and desmin were used as loading controls. The same concentration of protein was added to each lane for all experiments. One‐way ANOVA analyses, followed by an unpaired *t*‐test, were used to identify significance.

We confirmed our in vitro data by analyzing the hearts from HF mice for pCx43. There was clear evidence of hyperphosphorylation of Cx43 at myocyte junctions within hearts from mice treated with Ang2/Iso compared with myocytes from control saline‐treated mice (Figure 8A–F). We also observed pCx43 located at the lateral sites of cell–cell contact. This re‐localization of gap junctions to lateral positions between cardiomyocytes has been shown to be a key phenotype leading to HF.^44^, ^45^, ^46^, ^47^ Interestingly, Cx43 junctions between cardiomyocytes from mice containing Ang2/Iso pumps + weekly injections of CTP‐miRNA106a showed levels of phosphorylation similar to those found in untreated mouse hearts. Image J analysis of cardiomyocytes from sectioned hearts identified hypertrophic cardiomyocyte growth in hearts from mice containing Ang2/ISO‐pumps compared with controls (Figure 8G). CTP‐miRNA106a‐treated mice showed evidence of hypertrophic rescue. Picrosirius Red‐staining of treated and control hearts further identified partial rescue of collagen secretion by CTP‐miRNA106a (Figure S6). Additionally, Western blot analyses showed that all of the protein expression observed in vitro in control, Ang2/PE‐treated, and Ang2/PE+CTP‐miRNA106a‐treated hCMs were replicated in vivo (Figure 8H).

## 4 DISCUSSION

This study is a first‐of‐its‐kind showing that a miRNA cargo can be delivered specifically to the heart using a CTP. Previously,^4^ we identified that once delivered to hCMs, CTP‐miRNA106a reversed hypertrophy by downregulating HF genes CaMKIIδ and HDAC4.^6^, ^21^, ^22^, ^23^, ^24^, ^25^, ^26^, ^27^, ^28^, ^29^, ^30^ Here, we identified the efficacy and bio‐distribution of CTP‐miRNA106a for rescuing hormonally‐induced HF in a mouse model. Our data showed that CTP delivered its miRNA106a cargo ‘specifically’ to the heart in 30 min, followed by clearance of CTP from the heart to the kidneys and, to a lesser extent, to the liver by 3–5 h post‐systemic injection of the drug. The delivered miRNA106a reversed Ang2/Iso‐induced hypertrophy in ∼90% of the experimental mice. The work presented here also focused on two mechanistic pathways that miRNA106a potentially targets to reverse hypertrophy: the PLCβ1 and NF‐κB pathways. PLCβ1 is a direct target for miRNA106a, while the NF‐κB pathway is indirectly targeted by decreased Camk2δ (an miRNA106a target) signalling to IκBα.^[^ ^48^, ^49^ ^]^ This two‐pronged, in vitro/in vivo approach establishes a thorough feasibility outcome/proof‐of‐concept for CTP‐miRNA106a mechanisms and its ability to reverse HF parameters in mice.

The evidence abounds in the literature validating numerous intracellular signalling pathways that, when misregulated, can lead to various phenotypes of HF.^10^ In a thorough review by Stansfield et al.,^10^ 10+ pathways are identified in the development of HF. One common inducer of those pathways is overactive neurohormonal signalling, which includes the RAAS and the adrenergic pathway. To date, the HF intracellular signalling hubs that have been identified through accumulating bodies of literature include the CamK2δ pathway.^4^, ^24^, ^25^, ^28^ The PLCβ1 pathway,^10^, ^50^ the JakStat3 pathway,^51^, ^52^ the cyclic adenosine monophosphate (cAMP) pathway,^53^ and the mitogen‐activated kinase pathway (MAPK),^10^ at normal levels/activity, are necessary for maintaining homeostatic physiology and preserving anatomy within cardiomyocytes. However, once these pathways undergo sustained activation in response to various factors (e.g., high blood pressure, myocardial ischemia/infarction, metabolic stress, low cardiac output states, etc.), they lead to constitutive RAAS and catecholamine release, which induces adverse remodelling of the heart and ultimately, HF. Pharmacological agents blocking Ang2 and norepinephrine (ARBs, ACE inhibitors, beta‐blockers, etc) have been used clinically for decades as treatment for established HFrEF; however, this approach still inevitably results in worsening and progression of HF leading to substantial morbidity and mortality worldwide. As a result, quieting overexpression of genes and their translational products within one or multiple signalling hubs, specifically in cardiomyocytes, is potentially a compelling strategy for halting the pathophysiology culminating in the progression of HF. Interestingly, miRNA106a is predicted to target genes in all of these pathways (Table 3; Figure S7).^54^, ^55^, ^56^

PKC has been targeted using various inhibitors, many with success at reversing HF in most animal models of HF; however, this success has not been duplicated in humans. Weeks and McMullen^13^ qualified the current discordant human data as possible species differences, dosage effects, or timing and duration of PKC inhibition/reduction. Based on our findings, we would argue that suppressing PKC activity may only attack half of the bifurcated pathway. Alternatively, by going upstream and targeting PLCβ1 with CTP‐miRNA106a, and suppressing both IP3 and PKC activity, multiple signalling sources of HF are influenced and suppressed. For example, perpetual activation of PKC upregulates the signalling cascade leading to phosphorylation of downstream targets such as the gap junction protein Cx43, decreasing its conductance function.^41^, ^42^, ^44^ We found that CTP‐miRNA106a prevented chronic Ang2/PE‐induced Cx43 phosphorylation by knocking down PLCβ1, resulting in decreased PKC activity.

We were also curious if CTP‐miRNA106a could affect pathways that drive the inflammation arm of HF. This curiosity arose because CamKIIδ has been identified as a kinase that can phosphorylate IκΒ−α, resulting in its degradation and, thus, activation of the NF‐κB pathway.^49^ We surmised that if miRNA106a could downregulate CamkIIδ, then IκΒ‐α levels should return to normal, resulting in re‐establishment of homeostatic NF‐κB signalling. NF‐κB is a prime driver of cytokine production, such as IL‐1β.^57^, ^58^ Interestingly, the combination of immunofluorescence data, Western blots, NF‐κB response element data, and activation of downstream NF‐κB genes provided strong evidence that CTP‐miRNA106a could target signalling pathways involved in the inflammation arm of HF.

Gain‐of‐function analyses have reported that over‐expression of CamkIIδ mediates fibrosis by way of inflammatory gene expression and inflammasome activation in cardiomyocytes, while loss‐of‐function analyses have shown significantly reduced inflammatory gene responses in cardiac‐specific CamkIIδ knockout mice.^48^, ^59^ In both cases, it was found that CamKIIδ was responsible for attenuating the NF‐κB signalling cascade. That said, our data raises the possibility that CTP‐miRNA106a could represent a change of approach for treating HF by targeting inflammation, along with the other pathways detailed above.

In contrast to our work, Guan et al.^60^ presented data suggesting miRNA106a induced hypertrophy by targeting and down‐regulating mitofusin 2 (Mnf2). Unfortunately, we could not find transfection concentrations or specified culture times for miRNA106a mimics or inhibitors used in a study by Guan et al.^60^ making it impossible to duplicate their work. Our prior findings are in diametric contrast, showing abrogation of hormonally induced hypertrophy by miRNA106a in vitro^4^ and in the current work in vivo. The in vivo data in the study by Guan et al.^60^ appeared to show a correlation between inhibition of miRNA106a and reversal of cardiac hypertrophy; however, they injected into mice a non‐specific, cholesterol moiety‐linked miRNA106a inhibitor, which made it unclear if the miRNA106a inhibitor was actually delivered to the heart. It is possible that their cardiac results could have been due to inhibiting miRNA106a in other organs or tissues, such as in vascular endothelial cells, where vasodilator‐stimulated phosphoprotein (VASP) is a predicted target of miRNA106a. Misregulation of VASP in endothelial cells^61^ could alter vessel tone, resulting in changes in cardiac physiology and morphology as reported in the study by Guan et al.^60^

Based on our in vitro and in vivo data, we believe CTP‐miRNA106a could address an unmet medical need for delivery of a molecular therapeutic to treat HF that goes directly to cardiomyocytes and acts on HF hub signalling pathways such as CAMKIIδ, PLCβ1, and NF‐κB pathways. Attacking multiple hubs at once using a miRNA may be a better approach for treating HF than a one‐hub‐at‐a‐time approach, as is currently done with pharmacological agents, which have not solved the problem even at the highest of dosages. Alternatively, attacking the production of protein at its source, the mRNA(s), could prevent or reverse remodelling by returning overactive HF intracellular signalling hub proteins back to normal, homeostatic levels. This treatment could potentially be prescribed to newly diagnosed HF patients in whom cardiac hypertrophy and/or inflammation are playing a central pathophysiological role. CTP‐miRNAs would be a first in its class, a cardiac‐targeted heart failure drug, leading to a lower risk profile for populations with heart failure.

We recognize the limitations of our study. While we used a neurohormonal mouse model of HF, there are several models available, each with its own limitations, and none is an exact replica of human disease. We also did not report on blood pressure changes/rescue by CTP‐miRNA106a in this report, as this parameter is being collected and analyzed for a future study. Also, limiting, although the hCM cell line used here was verified to be cardiac cells with no contamination from cardiac fibroblasts or endothelial cells by RNA sequencing, RT‐qPCR, Western blot analyses, and immunofluorescence analyses in previous studies,^36^ it is still a cell line, and the data should be interpreted as such. However, our in vivo studies detailing the ability of CTP‐miR106a to reverse hormonally induced hypertrophy suggest that we are on the correct track for identifying an alternate approach for treating HF by targeting the underlying pathophysiology, instead of simply ameliorating the after‐effects of these activated pathways. Based on the data, we conclude that CTP‐miRNA106a can rescue hearts within Ang2/Iso‐treated mice.

## AUTHOR CONTRIBUTIONS

G. Ian Gallicano conceptualized experiments and performed many experiments and provided a significant portion of the writing. Ming Lu performed most experiments and assisted in the bulk of the writing. Siqi Cai, Claire Deng, Kaan Taskintua, and Jack Lopuszynski performed key technical experiments including Western blotts, qPCR, and biodistribution studies. Kyle Korolowicz performed all ultrasound heart analyses. Bryn Schoonover performed key cell counting, cell size, and Picrosirius red‐staining of collagen in heart tissue. Hongkun Wang verified all statistics, Raymon Yurko and Kazi Islam performed all organic chemistry to produce the CTP‐miRNA106a drug. Gerard Ahern performed all calcium imaging experiments. Maliha Zahid, invented CTP and provided expertise and consultation for entire paper.

## CONFLICT OF INTEREST STATEMENT

Maliha Zahid and G. Ian Gallicano were funded, along with Vivasc, Inc., by the NIH with an STTR grant. The remaining authors declare no conflict of interest.

## Supporting information



Supporting Information
