# Supplementary material for: Reversing cardiac hypertrophy and heart failure using a cardiac targeting peptide linked to miRNA106a
Source: Clin Transl Med. 2025 Aug 17;15(8):e70432. doi: 10.1002/ctm2.70432 (PMC12358689; doi:10.1002/ctm2.70432)
Supplement: Supplementary file 1 — Supporting Information [file CTM2-15-e70432-s001.docx]

**Supplemental Figures**


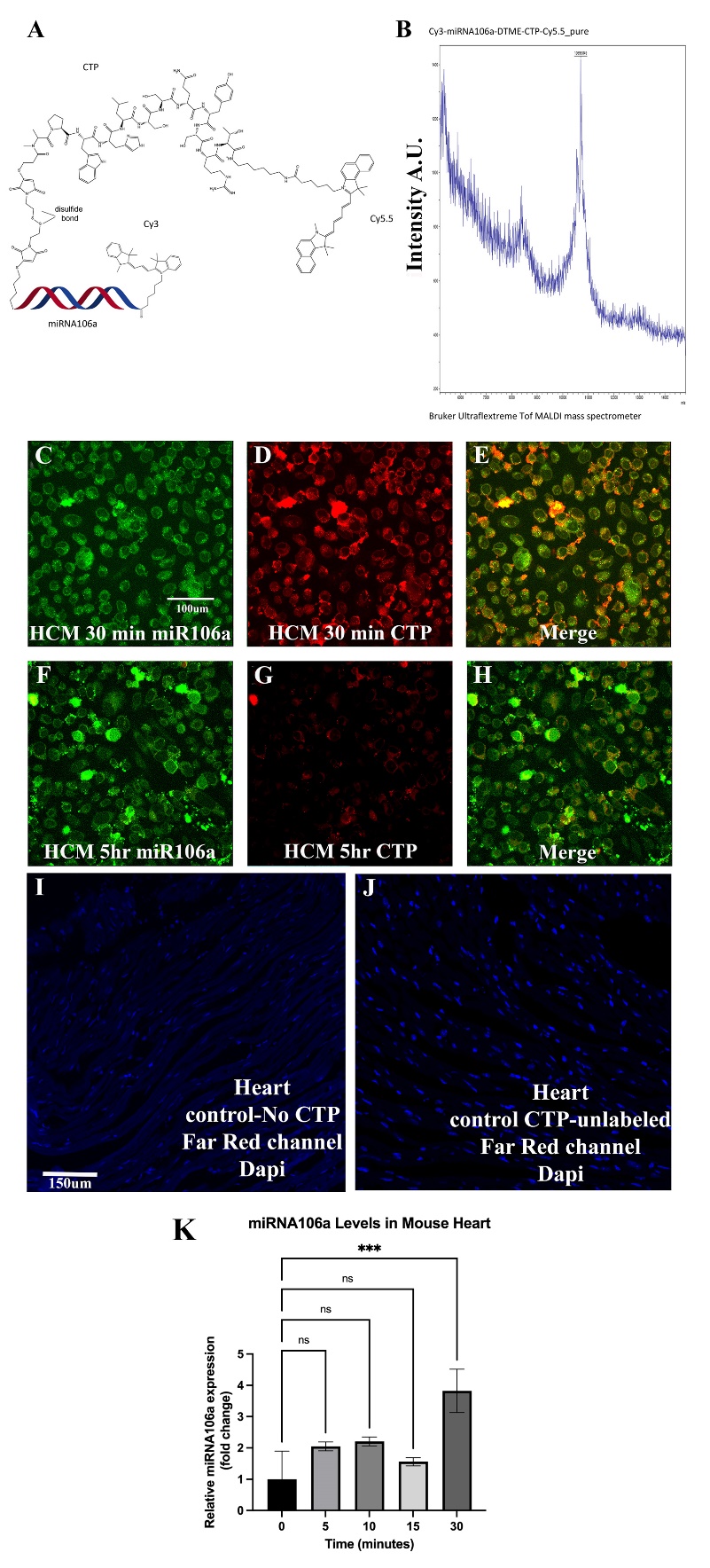


**Supplemental Figure 1**. The dual-labeled CTP-miRNA106a. **A**) Illustration showing the disulfide bond that is easily cleaved by endogenous disulfide reductases resulting in the CTP-Cy3 and the miRNACy5.5. **B**) Maldi-TOF analysis showing the correct molecular weight peak, which is predicted to be at 10700 Daltons. **C-H**) HCMs treated with the dual-labeled CTP-miRNA106a revealed delivery of miRNA106a to HCMs within 30min. The CTP signal that was also present at 30min was lost after 5hrs of incubation providing evidence that HCMs clear the CTP and keep miRNA106a.

**Supplemental Figure 1**. The dual-labeled CTP-miRNA106a. **A**) Illustration showing the disulfide bond that is easily cleaved by endogenous disulfide reductases resulting in the CTP-Cy5.5 and the miRNACy3. **B**) Maldi-TOF analysis showing the correct molecular weight peak, which is predicted to be at 10700 Daltons. **C-H**) HCMs treated with the dual-labeled CTP-miRNA106a revealed delivery of miRNA106a to HCMs within 30min. The CTP signal that was also present at 30min was lost after 5hrs of incubation providing evidence that HCMs clear the CTP and keep miRNA106a. **I-J**) Hearts from mice injected with unlabeled CTP (CTP alone) shows no autofluorescence. **K**) qPCR detects miRNA106a delivered by CTP at ~30min where significant expression is observed. *** p=0.0001 One-way ANOVA, followed by unpaired t-test to identify significance.


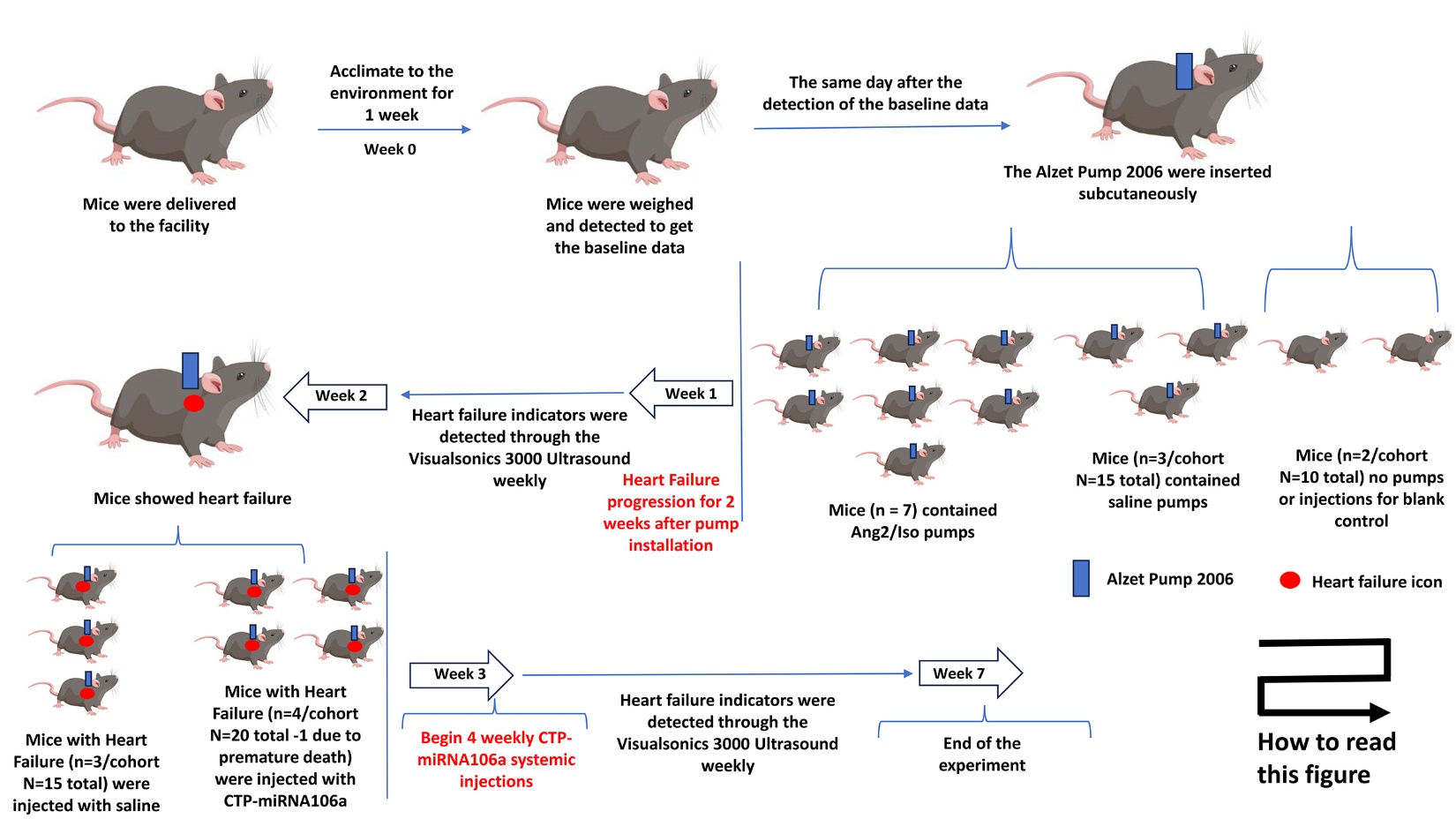


**Supplemental Figure 2**. Timeline illustrating the experimental setup and analyses of *in vivo* experiments. Note: *Two mice that had an Ang2/Iso pump died in the hands of the injectionist during the injection of CTP-miRNA106a. Upon necropsy, we found their hearts were significantly enlarged (e.g., Figure 3B). Veterinarians suspected they died of complications of the enlarged heart. Consequently, we included them in experiments such as EF, LV Mass, FS, immunofluorescence, Westerns blots etc. because their death was most likely attributed to their hormone-containing pumps. Also Note: one other mouse died one week after injection with CTP-miRNA106a. A necropsy showed enlarged heart but no other organ anomalies. It is suspected this mouse died of HF before the CTP-miRNA106a injection could take effect.*


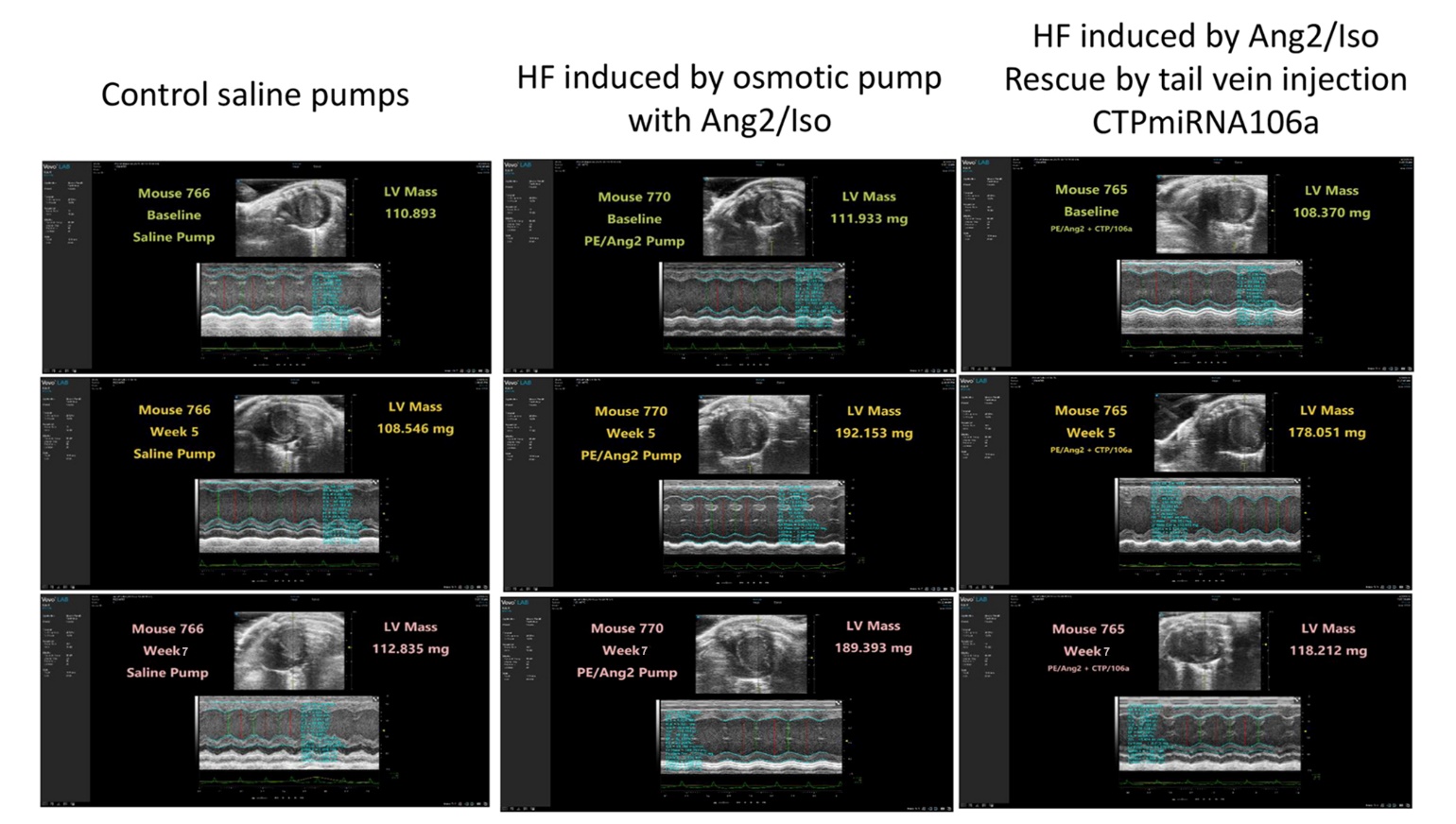


**Supplemental Figure 3**. Systemic injections of CTP-miRNA106a rescues Ang2/Iso-induced hypertrophy. Sonograms Showing LVMass from a typical experimental mouse cohort. ***Top row*:** Week 0. ***Middle row*:** Week 5. ***Bottom row***: Week 7. ***Left column***: Saline pump/saline injections results in no significant changes 0-7 weeks. ***Middle column***: Ang2/Iso pump/saline injections results in significant increase in LVMass. ***Right column***: Ang2/Iso pump/CTP-miRNA106a injections week 3-6 results in reversion/rescue of LVMass.


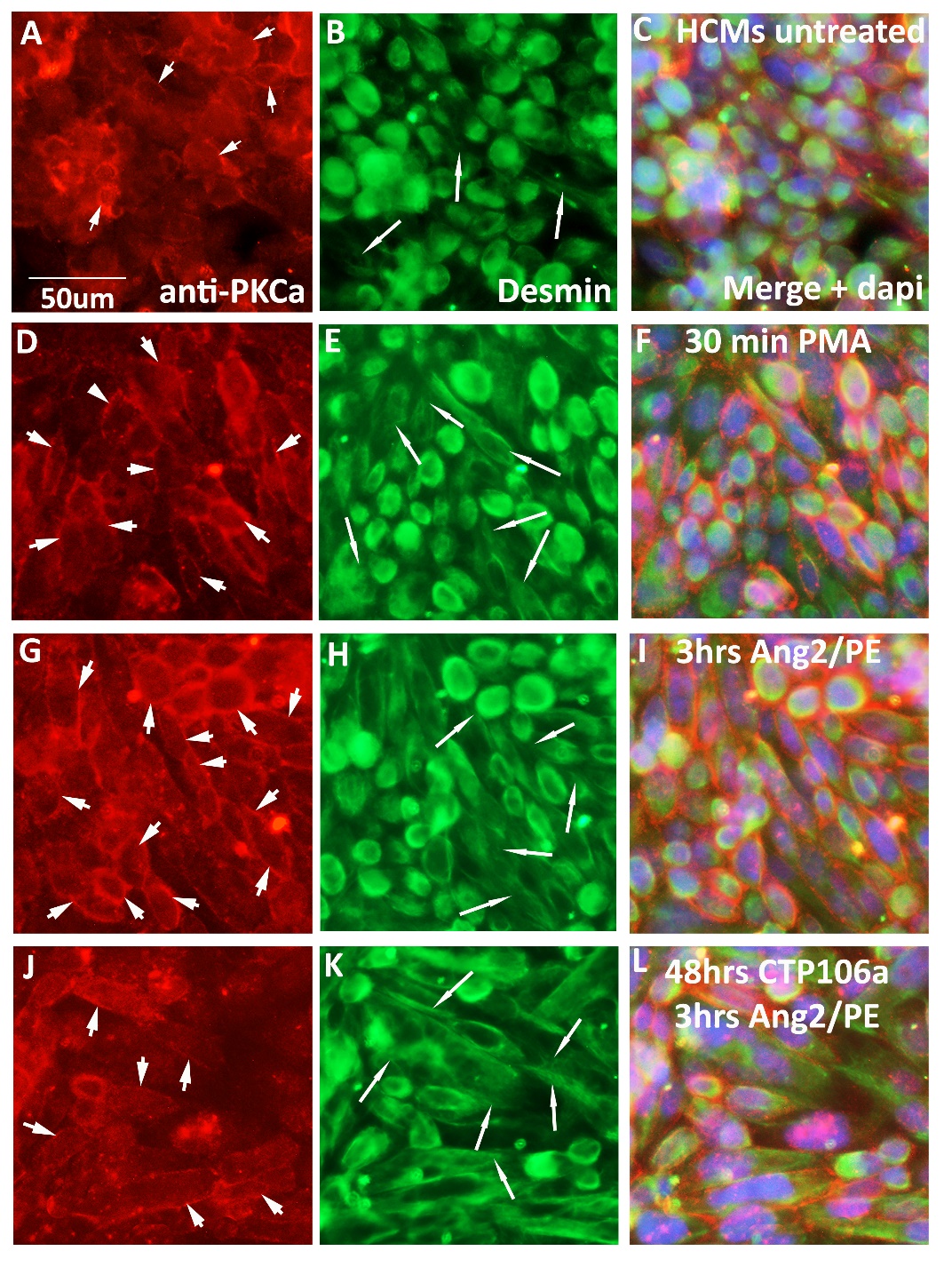


**Supplemental Figure 4**. Ang2/PE induction of PKC translocation to the plasma membrane is inhibited by CTP-miR106a. Translocation of PKC is required for its activation. **A-C**) Anti-PKCalpha was used to probe hCMs for PKC localization. Arrowheads point to a few cells showing membrane localization of PKC. **B)** Anti-desmin, the intermediate filament specific to cardiomyocytes in the heart (arrows point to whispy filaments) identifies these cells as hcms. **C)** The merged image shows more detail of nuclei (dapi blue), PKCa, and desmin localization. **D-F**). Phorbol myristate acetate (PMA), a synthetic activator of PKC, induces localization of PKC to the plasma membrane after 30 min in most hcms (Arrowheads). **G-I).** Ang2/PE causes translocation of PKC to the plasma membrane (arrowheads); however, **J-L**) shows that CTP-miR106a can prevent PKC translocation to the plasma membrane. Arrowheads point to a few cells with membrane staining. Quantification of membrane-bound PKC in **Table 2**. N=3 experimental replications


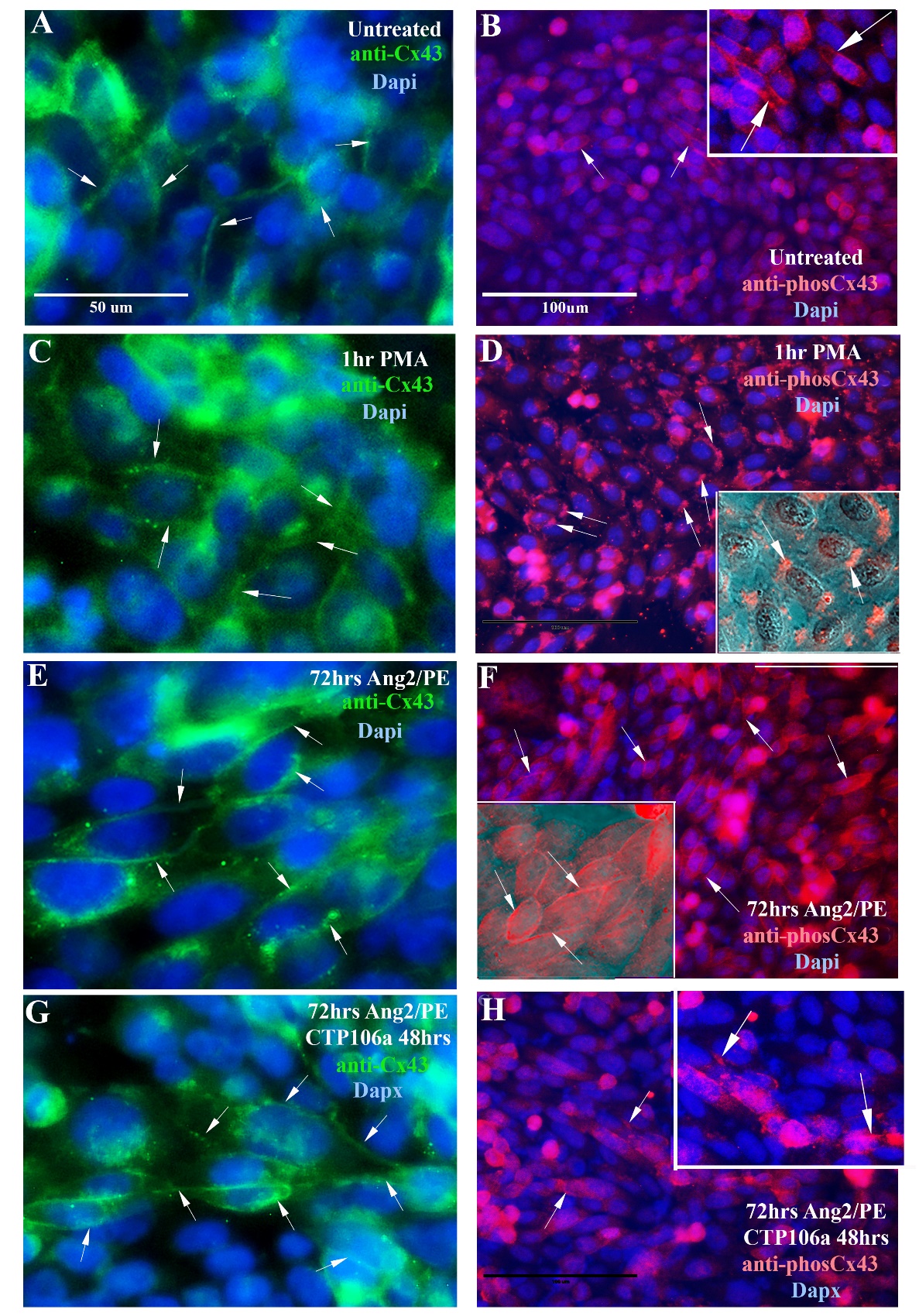


**Supplemental Figure 5**. One downstream target of PKC activity in hCMs is the gap junction protein Connexin 43 (Cx43). CTP-miR106a decreases Ang2/PE induced Cx43 phosphorylation. **A-B**) Gap junctions are prevalent in untreated, confluent hCMs (arrows in **A**); however, only a few pCx43-positive junctions (arrows in **B** and higher magnification **inset**) are observed using an antibody directed specifically to the PKC phosphorylated serine (Ser368). **C-D**) The phosphorylation pattern changes becoming more robust at junctions (arrows in **D** and higher magnification **inset**) between hCMs that are incubated in the PKC activator PMA for 1hr. Arrows in high magnification inset point to Phospho-Cx43 positive junction between cells. A phase contrast image is shown in the inset to show the gaps junction spatial relationship to the nucleus within each cell. Dapi was not used because Nuclei are clearly visible in these Phase images. **E-F**) hCMs incubated in Ang2/PE for 72hrs show a robust, widespread pCx43 pattern between and surrounding many hCMs (arrows in **F** and high magnification **inset**). **G-H**. While gap junctions remain intact (arrows in **G**), hCMs treated with Ang2/PE, followed by CTP-miR106a result in loss of phosphorylated Cx43. Quantification of pCx43 positive hCMs found in **Table 2.** N=3 Experimental Replications


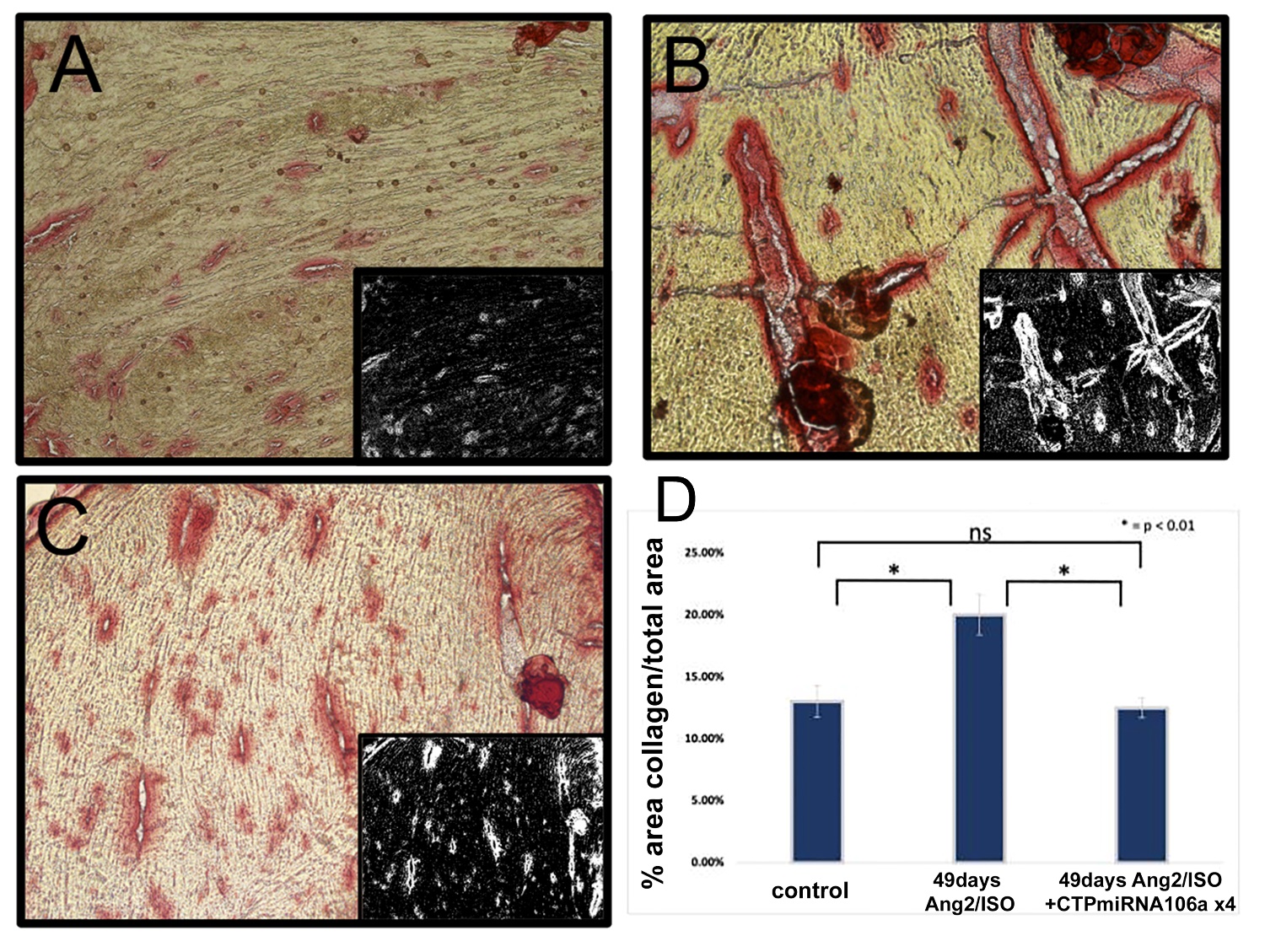


**Supplemental Figure 6.** Representative Picrosirius Red-stained images of control (A), heart failure (B), and rescued, CTP-miRNA106a-injected (C) ventricular tissue capture at 10X magnification. Collagen-rich areas appear red with staining and indicate fibrotic lesions. The inset images depict the binary masks utilized to conduct quantitative pixel analysis. White areas represent collections of pixels with an RGB score within the parameters set. Quantitative analysis identified the heart failure tissue as the largest collagen-positive area relative to the total tissue area. One-way ANOVA identified that the heart failure and rescued tissues had a significant (p<0.01) difference in the collagen-positive area; however, the control and rescued hearts were not significant relative to each other, suggesting fibrosis recovery.


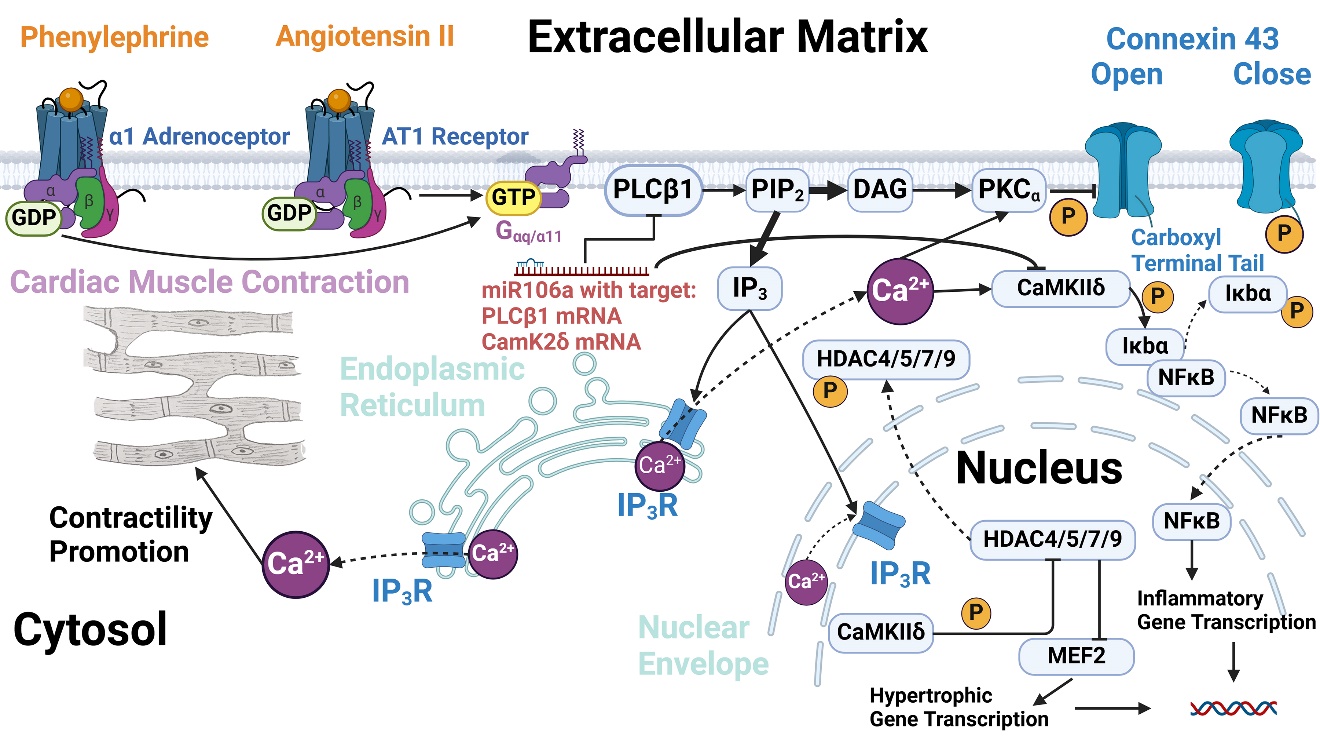


**Supplemental Figure 7**. Illustration of the pathway being tested. Angiotensin II-induced cardiac hypertrophy through Gq/PLCβ1/PKCα pathway (ATR: angiotensin II receptor, IP_3_: inositol 1,4,5-triphosphate, IP_3_R: IP_3_ receptor, PIP_2_: phosphatidylinositol 4,5-biphosphate, CaMKIIδ: Calcium calmodulin-dependent protein kinase 2 delta, HDAC4: histone deacetylase 4, NFAT: nuclear factor of an activated T cell. Illustration created using BioRender.com [62].

**Supplemental Materials and Methods**

*CTP-miRNA106a production*

Briefly, a C6 protected miRNA oligomer is dissolved in Triethylammonium bicarbonate (TEAbc) and reduced to a free thiol using DL-Dithiothreitol. The sample is centrifuged to allow for isolation of the miRNA pellet after two isopropanol extractions at 4°C. The pellet is then dissolved in NaOAc/35% acetonitrile and then reacted with 40eq. dithio-bis-maleimidoethane (DTME) and lyophilized to a dry powder overnight. Resuspension of the lyophilized miRNA-DTME intermediate in nuclease free water is followed by centrifugation of the sample at 6,000 rpm to isolate the resulting miRNA-DTME pellet from unreacted DTME. The DTME-miRNA pellet is reconstituted in NaOAc/10% acetonitrile and allowed to react with 10eq of thiol containing CTP peptide. The resulting miRNA-DTME-CTP conjugate is HPLC purified followed by lyophilization and then re-lyophilization from nuclease free water. MALDI analysis of the purified conjugate is then used for confirmation of the expected mass and to identity the final miRNA-DTME-CTP product.

*Production of Cy5.5-CTP-miRA106a-Cy3 dual fluorescent probe*

Dual, fluorescently-labeled CTP-miRNA106a is synthesized with an intervening disulfide bond for tracking studies. Synthesis of the Cy5.5-CTP-miRNA106a-Cy3 probe begins with Solid phase peptide synthesis (SPPS) on THR-2-Chlorotrityl resin using Fluorenylmethyloxycarbonyl (FMOC) chemistry and Ethyl-(*2Z*)-2-cyano-2-hydroxyiminoacetate/*N,N Diisopropylcarbodiimide* (Oxyma/DIC) activation on a Liberty CEM microwave synthesizer. After completion of peptide chain assembly, the free N-terminal amino group of the fully protected CTP peptide resin is manually conjugated with 3-(Tritylthio)proprionic acid using *N,N,N′,N′*-Tetramethyl-*O*-(benzotriazol-1-yl)uroniumtetrafluoroborate/1-Hydroxybenzotriazole hydrate/Diisopropylethylamine/Dimethylformamide (TBTU/HObt/DIEA/DMF). Cleavage of a side chain protected Trt-CTP-CO_2_H peptide fragment from the 2-chlorotrityl solid support is accomplished using 20% 1,1,1,3,3,3 Hexafluoro-2-propanol (HFIP) in methylene chloride followed by solvent removal by rotary evaporation. The carboxy-terminus of the Trt-CTP-CO2H fragment is then activated with DIPCDI/DMF and reacted with Cy5.5-amine (Lumiprobe Corporation) overnight at room temperature. Final cleavage of the Trt-CTP-Cy5.5 intermediate with Trifluoroacetic acid (TFA):Thioanisole:Anisole:Ethanedithiol (90:5:2:3) is followed by precipitation in Diethyl Ether (EtO2). The resulting crude product is purified by semi-preparative C-5 RP-HPLC on a Waters Delta Prep 4000 chromatography system using standard Acetonitrile/0.1%TFA gradient conditions.

C6 protected miRNA106a-Cy3 oligomer (IDT technologies) is reduced to the free thiol form using DL-Dithiothreitol (DTT) in 0.1M Triethylammonium bicarbonate (TEAbc) at pH 8.5 and then reacted with dithio-bis-maleimidoethane (DTME) in 300mM NaOAc/acetonitrile @ pH 5.2. Purification of the DTME-miRNA106a-Cy3 intermediate is then followed by reaction with purified SH-CTP-Cy5.5 peptide in 300mM NaOAc/acetonitrile @ pH 5.2 with gentle mixing at room temperature. C-18 RP-HPLC purification of the resulting Cy5.5-CTP-miRNA106a-Cy3 conjugate using trimethylamine acetate (TEAA)/Acetonitrile gradients on a Waters Alliance chromatography system is followed by lyophilization and re-lyophilization from nuclease free water. MALDI-Tof analysis of the purified conjugate on a Bruker Ultraflextreme mass spectrometer using 3-hydroxypicolinic acid (3-HPA) matrix in ammonium citrate allowed for confirmation of the expected mass and identity of the final Cy5.5-CTP-miRNA106a-Cy3 probe.

*Mouse model of HF*

Alzet pump 2006 was used for mice weighing >20g because it has a six-seven week delivery. Loading of pumps followed instructions that come with each set of pumps. For angiotensin II (AngII, 1.5mg/kg BW/min over six weeks) and isoprenaline (iso, 30 mg/kg/day for six weeks) treatment were used. This model has been shown to work well in mice [33-34]. An equal number of male and female animals were anaesthetized with isoflurane (~2% v/v in 100% O_2_, 1 L/min), and Alzet minipumps (2006) were implanted subcutaneously in the back of the animals. In each group, 10 animals were included in the study. Carprofen (5–10 mg/kg BW, Rimadyl, Pfizer, NY, USA) was injected subcutaneously immediately before pump implantation. If necessary, pain relief was repeated every 24 h for three days. Mice were sacrificed by cervical dislocation in isoflurane anesthesia. Hearts were removed, and weight was normalized to tibia length (HW/TL). Subsequently, the heart was divided for biochemical, molecular, and histological analysis.

*Ultrasound analyses*

Heart failure indicators were measured at the beginning, pre-CTP-miRNA106a Injection, and at the end of the experiment imaging using the VisualSonics VEVO 3100 ultra high-resolution ultrasound machine in the Georgetown-Lombardi Preclinical Imaging Research Laboratory under 1-2.5% isoflurane anesthesia. The animals were depilated using Nair™. The Left Ventricle (LV) of the heart was imaged using the MX550D ultrasound probe transmitting at 40MHz. Echocardiography of the LV was performed in both transverse and longitudinal views in B and M Modes. Vascular ultrasound was performed in both transverse and longitudinal views in B and M Modes. All echocardiographic analyses [e.g. ejection fraction (EF), left ventricle mass (LVmass), fractional shortening (FS), etc] were performed using VisualSonics proprietary quantitative analysis software Vevo LAB© version 5.8.2 as previously described [63].

*Picrosirius Red Collagen Staining*

Transverse cryosections of murine hearts were stained for collagen using the Abcam Picrosirius Red Stain Kit (Cat# ab150681, Lot# GR3383290-1) and the manufacturer's protocol. The stained sections were imaged using brightfield microscopy on an Olympus IX83 Motorized Inverted Microscope. Representative photos of each tissue were taken at both 10X and 20X magnification and analyzed using ImageJ software (NIH, version 2.16.0).

*Image Analysis of Collagen Content*

The 10X magnification images were analyzed using ImageJ to determine the area of collagen relative to total tissue area. The images were separated into RGB channels, and thresholds were set only to include collagen-stained areas. Thresholds were kept consistent for all images within each condition. The collagen pixel count was then expressed as a percentage of the total pixel area and plotted for each condition. One-way ANOVA and statistical analysis were conducted using VassarStats (VassarStats.net).

*Image Analysis of Cardiac Hypertrophy*

Immunofluorescence images of cardiomyocytes were examined utilizing ImageJ software to measure cardiomyocyte size and hypertrophy. The perimeter of each cell was drawn, and the size was calculated using Image J. The average cell size was calculated using every visible cell in the image. The results were plotted, and statistical analysis was conducted utilizing VassarStats.

*Immunofluorescence analyses*

## Cells were grown on 0.1% fibronectin coverslips, fixed in 4% formaldehyde, followed by permeabilization with 0.1% Triton 100, then blocked by 2% bovine serum albumin (BSA) and incubated with antibodies for NF-κB (sc-372, Santa Cruz Biotech), PKCα (ab32376, Abcam), Connexin 43 (138388, ThermoFisher), Phospho-Connexin 43 (YB3833997, ThermoFisher), PKCα (sc-8393, Santa Cruz), CnnT (PA5-95994, Thermo-Fisher), and Desmin (E-AB-70228, Elabscience) in 1:100 dilution for one hour, then washed for another hour. All secondary antibodies were Alexa Fluo Plus Antibodies from ThermoFisher. Dapi (1351303, BioRad) was added for 30 minutes to amplify nuclear staining. Rhodamine-Phalloidin-594 (A12381; Thermo-Fisher) was used for actin staining. Slides were viewed and analyzed using an Olympus Fluoview 500 four-channel laser scanning microscope. Cell counting, area, and membrane analyses were performed using Image J.

*Western Blot*

Cells were scraped and transferred into 200 ul Laemmli Sample Buffer (#1610737, BioRad), followed by a Bradford Assay using RC DC Protein Assay (#5000121, BioRad) to measure the protein concentration of each sample. 10ul of samples were loaded into 4-20% Precast Polyacrylamide gel (#4568096, BioRad) well. After SDS-PAGE, proteins were transferred to polyvinylidene fluoride membranes through the BioRad mini gel system and incubated at room temperature with primary antibody (1:500-1:2000) in a Blocking Buffer (#12010020, BioRad) overnight. The antibodies were as follows: PLCβ1 (ab182359, Abcam), IL1β (ab216995, Abcam), Connexin 43 (PA5-11632, ThermoFisher), Phospho-Connexin 43 (YB3833997, ThermoFisher), IκB (PA5-120122, ThermoFisher), NF-κB (sc-372, Santa Cruz Biotech), BNP (CSB-PA07924A0Rb, Cusabio), CaMK2δ (ab181052, Abcam), IL6 (ab214429, Abcam), Actin (sc-47778, Santa Cruz Biotech), Desmin (sc-23879, Santa Cruz Biotech). All secondary antibodies were from Jackson ImmunoResearch Laboratories Inc. Gels were treated with Enhanced Chemiluminescence (Ref: 34580, ThermoFisher), and bands were visualized using an ImageQuant LAS4000 Analyzer (General Electric). ImageJ quantified bands to calculate pixel intensities. The pixel intensities were normalized to untreated samples (0h).

*Enzyme-Linked Immunosorbent Assay*

Cells were lysed with 1 mL of lysis buffer (20 mM MOPS, 50 Mm β-glycerolphosphate, 50 mM sodium fluoride, 1 mM sodium orthovanadate, 5 mM EGTA, 2 mM EDTA, 1% NP40, 1 mM dithiothreitol, 1 mM benzamidine, 1 mM phenylmethanesulphonylfluoride, and 10 μg/mL leupeptin and aprotinin). ATP initiated the PKC-induced phosphorylation of the substrate stuck to the wells. A phosphor-specific substrate antibody was then incubated with the samples for one hour and washed with the washing buffer. A secondary antibody conjugated with HRP was then used in a 1:1000 dilution.

Secreted IL1β concentration change was discovered by an IL1β Human Elisa Kit (ADI-900-130A, Enzo Life Science Inc). The cell culture medium was harvested and stored at -20℃, and then a sandwich Elisa was performed on the samples.

Both results were visualized under 450 nm absorbance with a PerkinElmer Multimode Plate Reader Enspire 2300 Touchscreen by adding 3,3’,5,5’-tetramethylbenzidine (TMB) substrate for 10min followed by the stopping solution.

*RNA Extraction and Reverse Transcription Quantitative Polymerase Chain Reaction*

The reference genes were desmoplakin previously published (34). qPCR assays for identifying miRNA106a in cultured cells was performed using the all-in-one miRNA RT-qPCR detection kit (GeneCopoeia Inc.). The kit comes with a 3’ universal primer that when used in sync with the miRNA106a primer (5’- AAAGTGCTAACAGTGCAGGTAG-3’) identifies miRNA106a in cells.

Total miRNA was purified from mouse organs by the miRNeasy mini kit (Ref 1038703, Qiagen). Then, RT-qPCR assays for identifying miRNA106a were performed using the same all-in-one miRNA RT-qPCR detection kit above. The miRNA106a primer for mouse organs is 5’-CAAAGTGCTAACAGTGCAGGTAG-3’.

All RNA concentrations were quantified using a nanodrop and normalized to 50 ng/μl before reverse transcription. All qPCR results were calculated with the delta-delta Ct method and normalized to the untreated group (0h) after normalizing to the U6 house keeping gene.

*FACS Analyses*

hCMs were left untreated or incubated with Ang2/PE for 24 hours or treated with Ang2/PE and CTP-miRNA106a 24hrs later and then prepared for FACS, which consisted first with incubation in 1000x diluted Brefeldin A (5ug/ml-Abcam Inc. ab193369) for 6hrs prior to fixation to inhibit secretion. hCMs for flow cytometry were fixed in 2% formaldehyde followed by permeabilization with 0.1% Triton-X-100. They were washed in PBS, blocked with 2% BSA in PBS, and then incubated in IL-1β antibody (ab216995, Abcam) overnight. The next day, hCMs were then incubated with Alex Fluor 488 secondary antibody for one hour. Flow cytometry data were acquired and analyzed by members of the Georgetown University Flow Cytometry Core Facility using a BD LSRFortessa Cell Analyzer, the Cat. No. is 647177.

*Statistical analyses*

One-way ANOVAs were performed on all data sets where more than two groups were analyzed. When significance (p<0.05) was identified, unpaired t-tests were used to identify significant group comparisons. These analyses were used to compare all band densities for Western Blots, delta-delta CTs for RT-qPCRs, IL1β concentration assay for ELISA, and heart failure indicator changes for mouse works. The Mann-Whitney U test was used to compare the PKC activity assay results. A Mann-Whitney U test compares the differences between two independent samples when the sample distributions are not normally distributed, which was shown in PKC assay results. The mean statistical comparison for each group included comparing untreated samples to Ang2/PE treated samples, Ang2/PE treated samples to CTP-miRNA106a treated samples, and untreated to CTP-miRNA106a. All results were analyzed and graphed using IBM SPSS and Prism. N>3 were the minimum measurements. A Kaplan Meier Survival Estimator was depicted to describe the probability that an individual mouse would survive beyond the injection of Ang2/PE alone and Ang2/PE/CTP-miRNA106a.

Additional Westerns

**Additional Westerns for Figure 1**. Westerns are loaded in the same order as shown in Figure 1. Each lane is a separate sample. Total Samples (n values) are as follows:

Untreated (0) n= 3; 24hrs n=3; 72hrs n=3; 144hrs n=3

Prevention of each hypertrophic marker n=9

Reversal/rescue of each hypertrophic marker n=13

CTP alone n=9


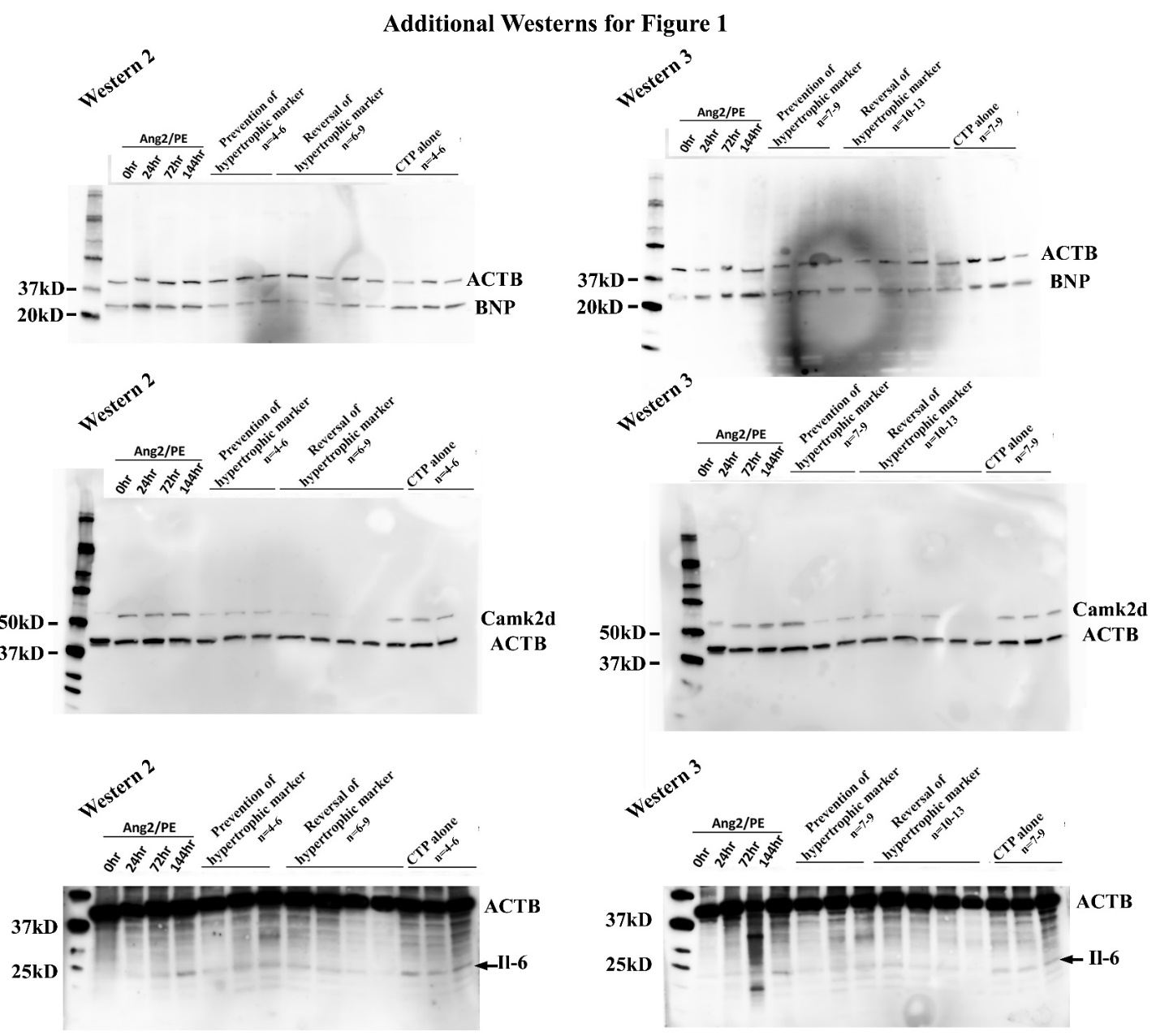


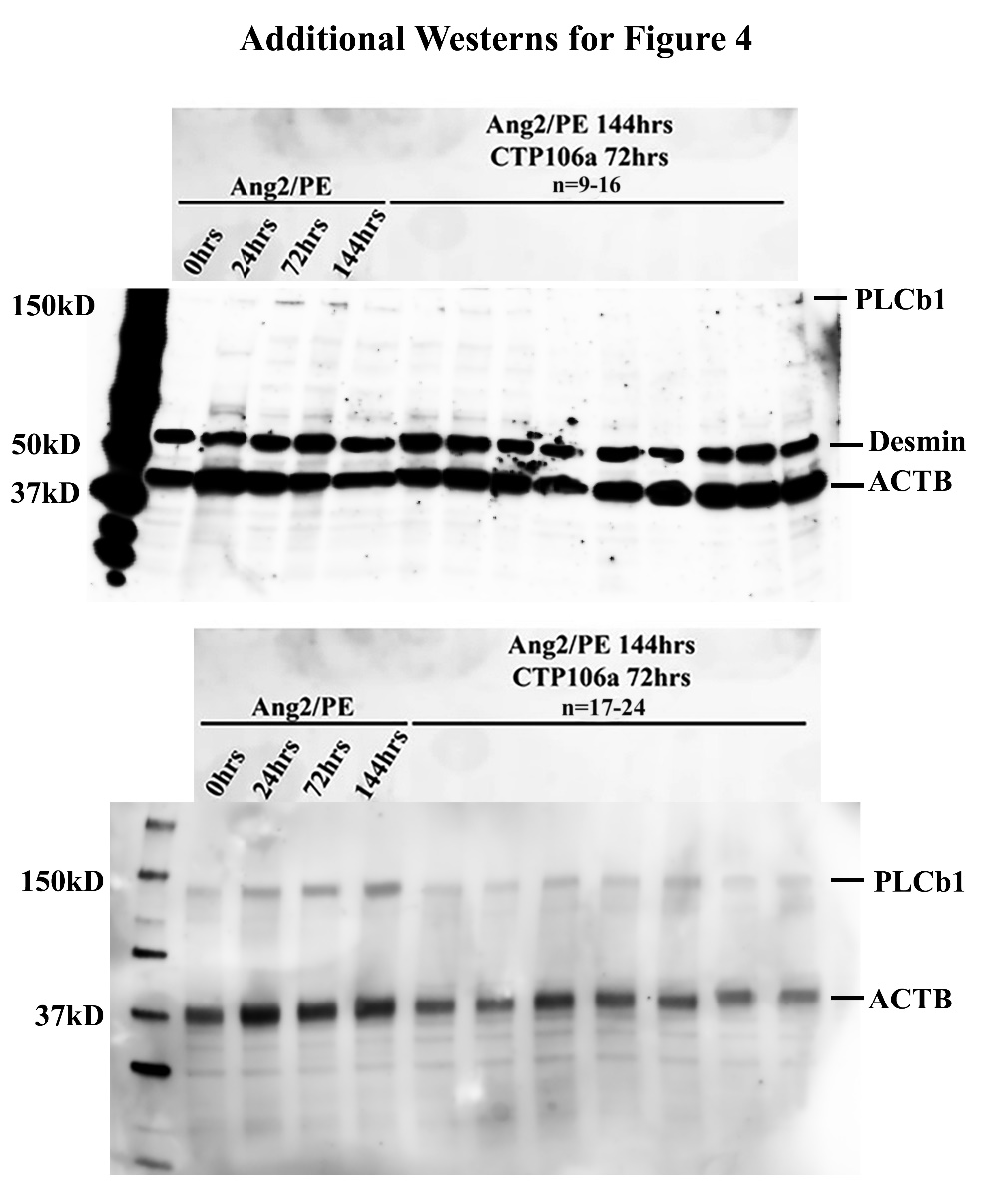


**Additional Westerns for Figure 4.** Westerns are loaded in the same order as shown in Figure 4. Each lane is a separate sample. Total Samples (n values) are as follows:

Untreated (0) n= 3; 24hrs n=3; 72hrs n=3; 144hrs n=3

Reversal/rescue of each hypertrophic marker n=24


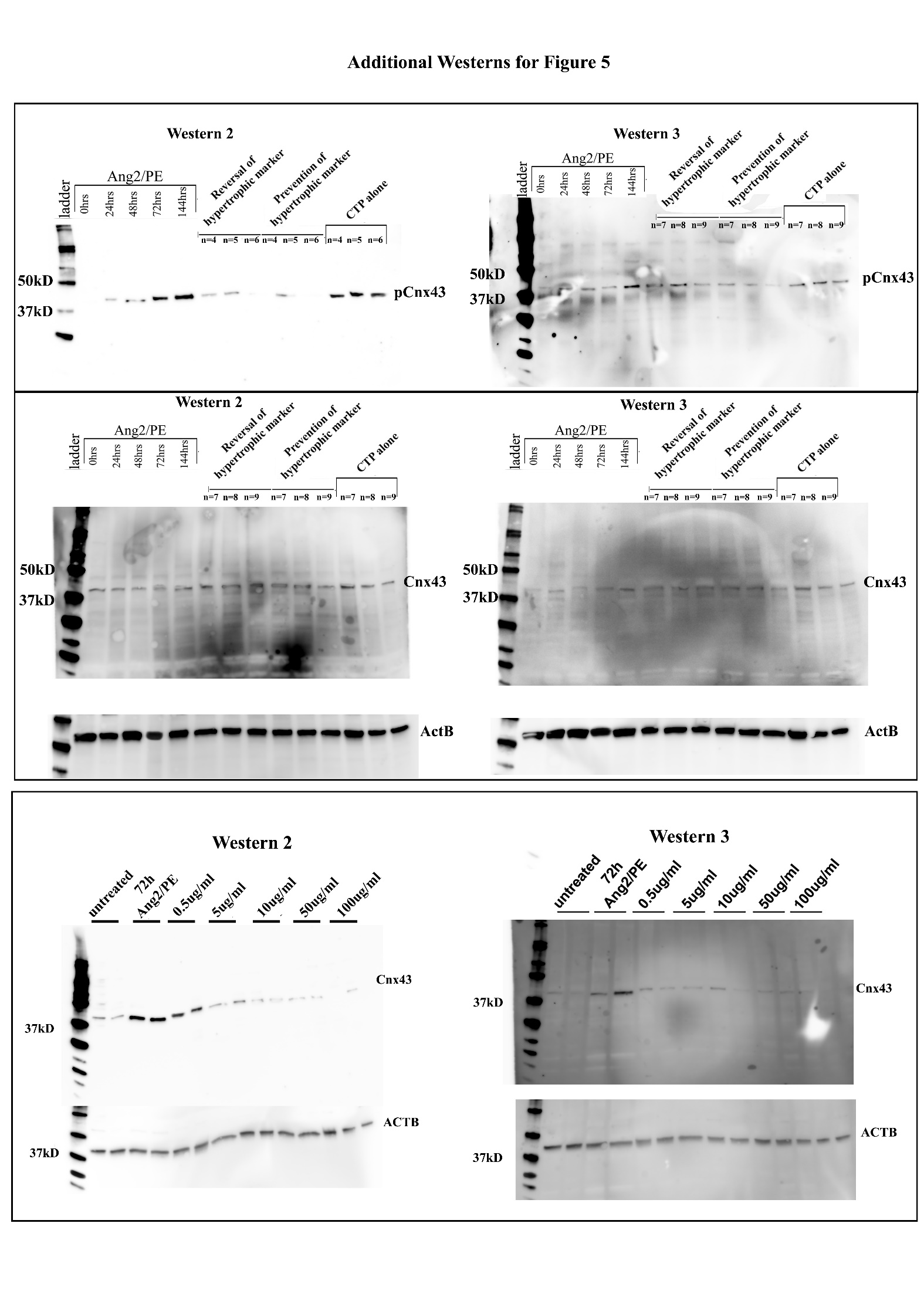


**Additional Westerns for Figure 5**. Westerns are loaded in the same order as shown in Figure 5. Each lane is a separate sample. Total Samples (n values) are as follows:

Untreated (0) n= 3; 24hrs n=3; 72hrs n=3; 144hrs n=3

Prevention of each hypertrophic marker n=9

Reversal/rescue of each hypertrophic marker n=9

CTP alone n=9

Dosage Westerns for Figure 5 are loaded similar to the Western in Figure 5I. Each lane is a separate sample

Untreated n=6; 72hr Ang2/PE n=6; n=6 for each treatment


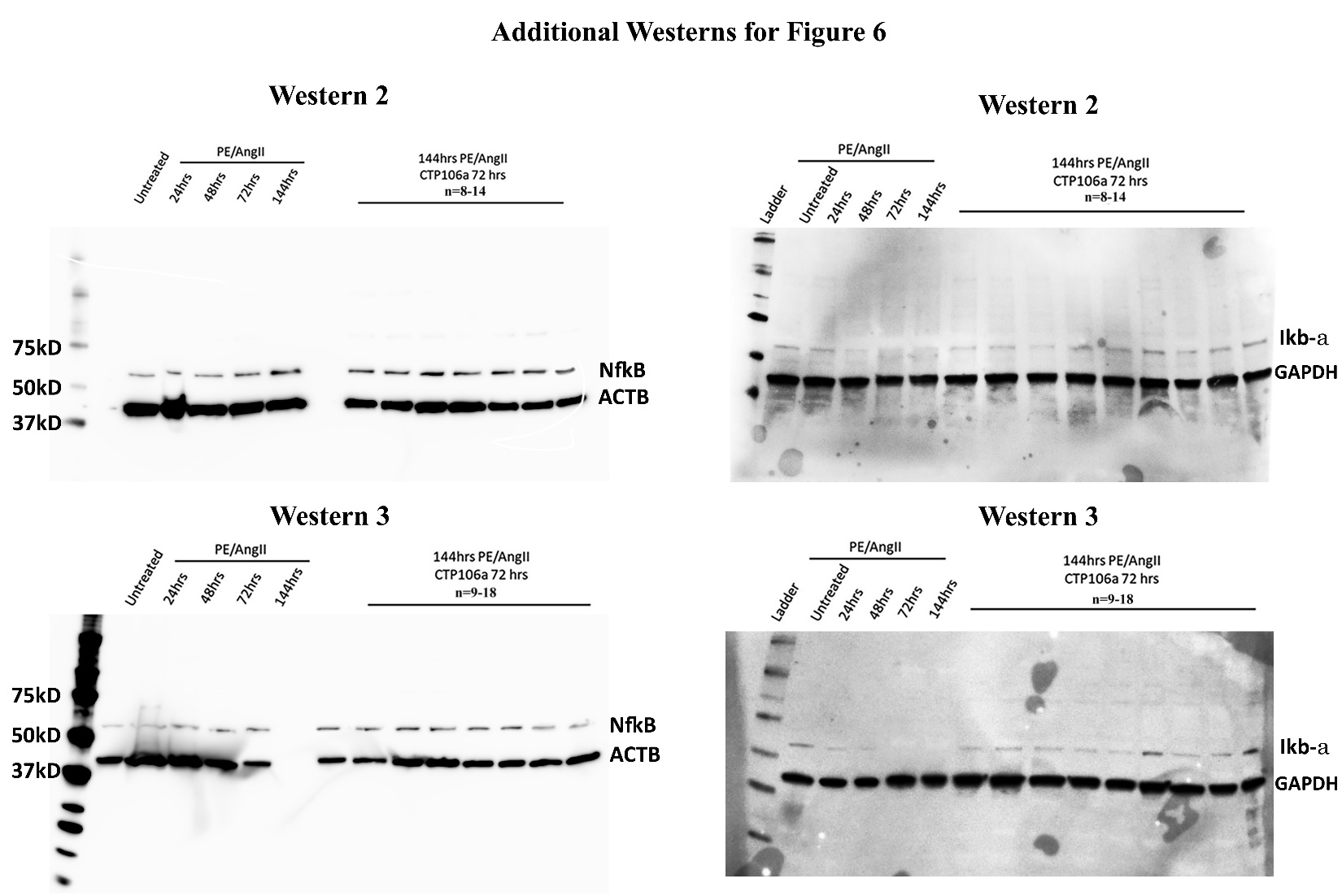


Additional Westerns for Figure 6. Westerns are loaded in the same order as shown in Figure 6. Anti NF-κB left, Ikba right. Each lane is a separate sample. Total Samples (n values) are as follows:

Untreated (0) n= 3; 24hrs n=3; 48hrs n=3; 72hrs n=3; 144hrs n=3

144hrs PE/Ang followed by CTP-miRNA106a (Reversal/rescue) n=18

CTP alone n=9


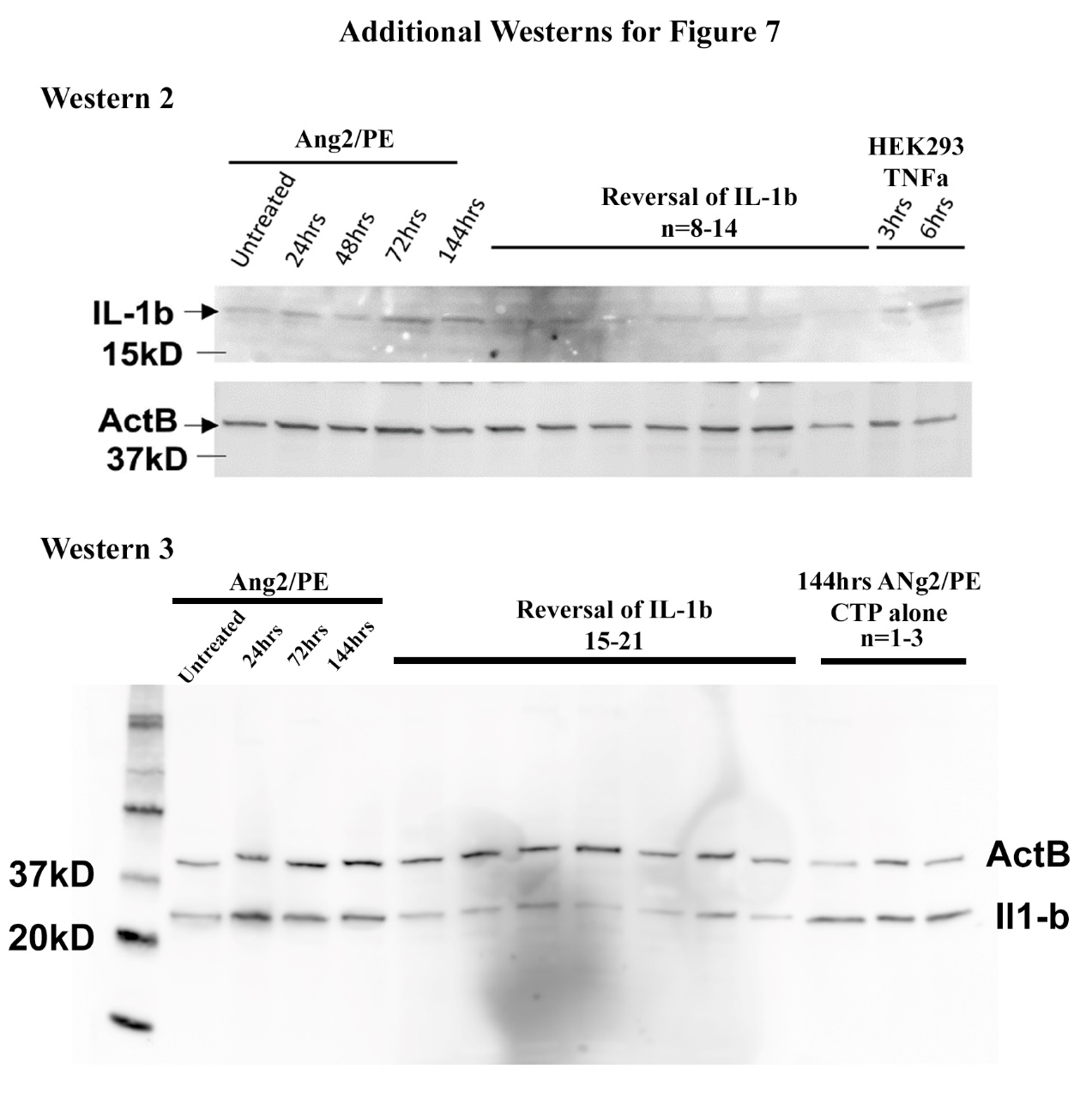


**Additional Westerns for Figure 7.** Westerns are loaded similarly as shown in Figure 7 with the exception of adding CTP alone after Ang2/PE. Each lane is a separate sample. Total Samples (n values) are as follows:

Untreated (0) n= 3; 24hrs n=3; 48hrs n=3 (from other westerns); 72hrs n=3; 144hrs n=3

144hrs PE/Ang followed by CTP-miRNA106a (Reversal/rescue) n=21 (including n=7 figure in main text)

CTP alone n=3


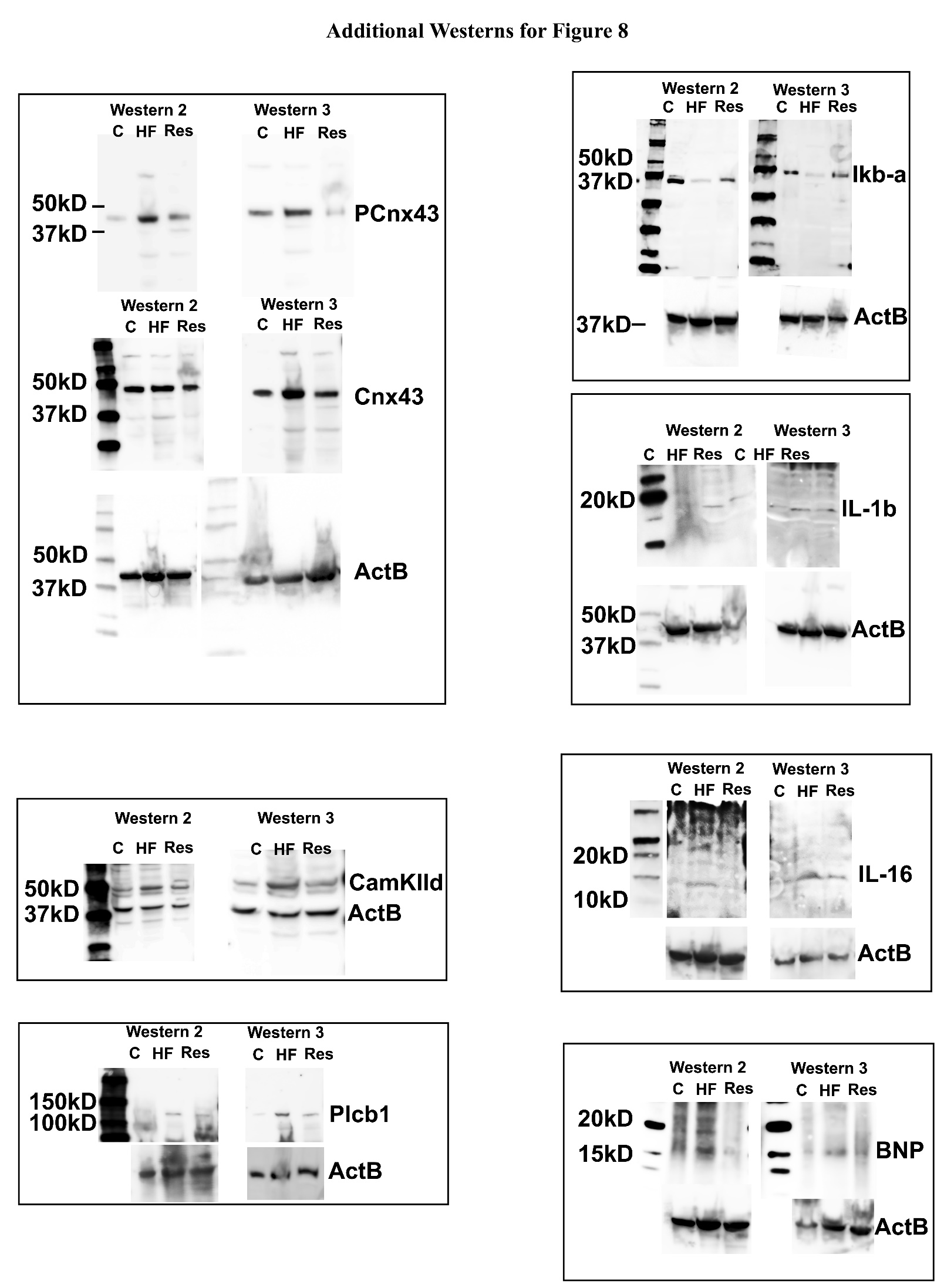


Additional Westerns for Figure 8. Westerns are loaded similarly as shown in Figure 8 with the exception of adding Actin for each Western. Each lane is a separate sample. Total Samples (n values) are as follows:

control (C) n= 3; 24hrs n=3; 48hrs n=3 (from other westerns); 72hrs n=3; 144hrs n=3

144hrs PE/Ang followed by CTP-miRNA106a (Reversal/rescue) n=14

CTP alone n=3
